# Graphical Discrimination of New Zealand Honey from International Honey Using Elemental Analysis

**DOI:** 10.1007/s12011-023-03680-6

**Published:** 2023-04-29

**Authors:** Megan N. C. Grainger, Hannah Klaus, Nyssa Hewitt, Han Gan, Amanda D. French

**Affiliations:** 1 School of Science, University of Waikato, Private Bag 3105, Hamilton, 3240 New Zealand; 2 Department of Mathematics, University of Waikato, Private Bag 3105, Hamilton, 3240 New Zealand

**Keywords:** Honey, Trace elements, Metals, Fingerprint, Geographical origin, Graphical discrimination

## Abstract

This study investigated the potential of utilising the elemental fingerprinting of honey to differentiate New Zealand (NZ) honey from that of international origin. Twenty elements were analysed by ICP-MS in 352 honeys from 34 various countries. Of these, 323 honeys (245 New Zealand honeys, 78 international) and two subsets of data (NZ and European origin, *n* = 306, and, NZ and Denmark/Germany, *n* = 280) were visualised using principal component analysis (PCA). For the NZ/Europe subset, 42.2% of data was explained in the first two principal components. Statistical classification rules were also derived using linear discriminant analysis (LDA) and decision tree analysis. Various combinations of elements were explored for classification, considering the effect of soil-derived elements and those from anthropogenic sources. A high degree of accuracy (at least 90%) for the characterisation of New Zealand honey was observed for all statistical models, showing the robustness of these analyses. When using decision tree analysis to distinguish New Zealand samples from international samples, a tree with five terminal nodes (using Cs, Ba and Rb) was created with 92.4% accuracy. This work has demonstrated that elemental fingerprints of honey are a promising tool for categorising New Zealand honey from other geographical locations.

**Supplementary Information:**

The online version contains supplementary material available at 10.1007/s12011-023-03680-6.

## Introduction

Traceability of food has become an important topic globally over the past couple of decades [1]. Food traceability can reassure consumers on the geographical origin, quality and safety of purchased food. Determination of geographical origin can help to protect brands and may influence a buyer’s decision based on the source of the food product. One example is mānuka honey, derived from *Leptospermum scoparium*, which is a highly sought after product due to its non-peroxide antibacterial (NPA) activity. Mānuka honey with high activity sells for a premium price; in 2021, beekeepers were paid up to NZ$120/kg for mānuka honey, compared to up to NZ$6/kg for clover honey [2]. This high commodity product may be subjected to fraudulent activity, including labelling international honey as mānuka or blending mānuka honey with international honeys to stretch the product to gain revenue. Identifying the country of origin may help deter this type of activity since non-genuine products will not comply with the criteria set for a pure product from a particular geographical location.

Honey is produced when bees dehydrate nectar or secretions of plants, or excretions of plant-sucking insects on the living parts of plants (i.e., honeydew). There are few steps from hive to shelf since CODEX states that substances may not be added to honey. Therefore the elemental composition of honey is mostly influenced by the bees’ diet, and addition of elements after harvest should be minimal (especially compared to a product which undergoes multiple steps, such as salami and dairy products) [3]. However, the use of fertiliser or point source contamination within the foraging range of the bee may influence the elemental fingerprint. Honey primarily consists of sugar and water but contains small amounts of elements (0.02–1.03 g 100 g^–1^) [4]. Although there are reports that botanical origin may influence the elemental profile, [5–9] other research states that elemental analysis of honey from the same botanical origin but different geographical locations have different elemental signatures due to the influence from the environment [10].

Mineral elemental fingerprinting is a powerful technique to characterise food to a particular geographical origin and has previously been demonstrated for various foods, including soybean, [11] coffee, olive oil, wine and meat [12]. Elements are stable over time as they do not degrade or react and their presence in food sources predominately originates from the soil and are taken up into plants. Elements can be classified as nutrients (required for life, including alkaline and some transition metals), micro-essential (minor role for life, including rare earth elements) and toxic (detrimental to growth, including heavy metals). Careful consideration of the elements best suited for geographical fingerprinting is required. Due to the uptake of nutrients and micro-essential elements by plants from soil, these are good choices, whereas those introduced via anthropogenic activity (e.g. heavy metals) are not as useful due to their fluctuation across samples within a region and over time from a single location [13]. However, caution needs to be applied as some nutrients and micro-essential elements may also arise from anthropogenic activity; for example, Cu is reported to be correlated to Cd in regions of agricultural practice [14]. A recent study by Meister et al. found no correlations between elemental concentrations in honey and soil in a set of mānuka honeys from New Zealand [15]. This is in contrast with studies from other geographical origins and floral sources [16].

A number of techniques have been reported for quantitative analysis of elements in honey, including atomic adsorption spectroscopy (AAS) [17], inductivity coupled plasma atomic emission spectroscopy (ICP-AES) [18, 19], inductivity coupled plasma optical emission spectroscopy (ICP-OES) [20–23] and energy dispersive X-ray fluorescence (XD-XRF) [24, 25]. In recent years, the determination of elements has mainly been carried out by inductively coupled plasma mass spectrometry (ICP-MS) [26–29], which has a number of advantages over other elemental techniques, including quick simultaneous analysis of multiple elements, relatively cheap analysis, high sensitivity and low detection limits (often down to parts per trillion). ICP-MS is becoming more readily available, with commercial laboratories and universities often having at least one instrument. To illustrate the powerful amount of data that can be generated using ICP-MS, if 20 elements are simultaneously analysed by ICP-MS in 100 samples, 2,000 data points are produced in a short time (< 3 h). Multivariate statistical analyses, including principal component analysis (PCA), linear discriminant analysis (LDA) and cluster analysis (CA) are often applied to handle the vast amount of data. These techniques are powerful because multiple elements can be assessed together and either determine clustering of similar samples or determine discriminant functions to predict the origin of a particular sample [13].

A wide array of elements have previously been used for discrimination of honey, including Ca, Ba, Fe, K, Mg, Mn, Na, P, Rb and Zn [18, 20, 28, 30, 31]. It has been noted that elements in low proportion for each element group (i.e. alkaline, alkaline earth and transition metals) are more reliable for fingerprinting than those that are most abundant [13]. For example, Rb is suggested as a more useful element than Na and K from the alkaline group. While a number of studies report the concentrations of various metals in honey from countries across the world, these papers do not attempt to categorise honey by country. The majority of studies that categorise honey do so for regions within a single country. For example, a study of 200 black locust honeys from five regions in Croatia determined that Al, Fe and K were useful elements for categorising into regions [10]. Another study determined that certain elements were able to categorise Italian honey by industrial, urban and rural regions. [32]

While there are reports in the literature that address the elemental profile of honey for honey from regions in relatively close proximity within a single country, there is currently no literature demonstrating the ability to categorise honey from separate countries, and more importantly, no literature published to investigate the possibility of distinguishing New Zealand honey from international honey. This research aimed to i) determine elemental signatures for New Zealand and international honeys, and ii) discriminate and categorise New Zealand honey from international honey, independent of botanical origin, based on the elemental signatures. This work demonstrates the potential of elemental analysis as one tool to help protect the identity of New Zealand honey.

## Materials and Methods

### Chemicals, Reagents and Apparatus

A multi-element stock standard (10 mg kg^−1^, IV71-A) and single-element standards (1000 mg kg^−1^) for Na, Ca, P, S, K and Fe were purchased from Inorganic Ventures, Christiansburg, VA, USA. Certified reference material (CRM) ERM-CE278k (mussel tissue) was purchased from European Reference materials and SLRS-6 river water CRM was purchased from National Research Council Canada. Nitric acid (65%, analytical grade) and hydrogen peroxide (30%, analytical grade) were purchased from Merck and Univar (Ajax Finechem Pty Ltd) respectively. Type 1 water was distilled and deionised using a Millipore Milli Q Reference water purification system (18.0 mΩ resistivity). Polypropylene tubes (15 mL) were purchased from Greiner. Minisart Syringe Filters (0.45 µm, cellulose acetate) were purchased from Sartrious, Germany. An Ohaus dry heat block was used for heating samples.

### Honey Collection

A total of 352 honey samples were analysed in this research. New Zealand honey samples (*n* = 245) were either obtained utilising citizen science from single apiary sites with known GPS locations around North Island, New Zealand from 2010 – 2019 (*n* = 181) or purchased commercially (*n* = 64). purchase from 34 countries (Germany, 20; Denmark, 15; Greece, 9; Italy, 7; 5 from both Poland and UK; 4 from both India and Russia; 3 from each of Australia, Chile and Spain; 2 each from United States, Turkey, Niue, France, China, Belize, Austria and 1 each from Vietnam, Uzbekistan, South Africa, South America, Portugal, Morocco, Mongolia, Maldives, Madeira, Japan, Georgia, Finland, Croatia, Belgium, and Argentina). Honey was extracted by beekeepers and provided for analysis. All samples were of nectar origin and a combination of mono- and multi-floral botanical origins.

For discrimination and categorisation, only countries with at least 5 samples were used; these samples were all of European origin and are referred to as “Europe” in the text. Additionally, a subset of 35 samples containing honey from Denmark and Germany was also used.

### Sample Preparation

Honey samples were prepared for analysis using a previously published method [33]. Briefly, honey (200 ± 20 mg) was heated (50 °C, 30 min) then centrifuged (10 min, 3000 rpm) to draw sample to the bottom of tube. Nitric acid (0.2 mL) was added and tubes were heated in a heat block (80 °C, 60 min); Type 1 water (0.5 mL) was added to CRM samples before digestion for wetting. Samples were cooled before addition of hydrogen peroxide (0.1 mL) and further heating (80 °C, 60 min). Following cooling, Type 1 water was added by autopipette or bottle-top dispenser to give a known final volume. The final volume was approximately 6 mL and was calculated individually for each sample. All samples were centrifuged before ICP-MS analysis (10 min, 3000 rpm); CRM samples were also filtered through 0.45 µm cellulose acetate filters.

### Instrumentation

Elemental analysis was performed using an Agilent 8900 Triple Quadrupole Inductively Coupled Plasma Mass Spectrometer (Q-ICP-MS; Agilent Technologies, Santa Clara, California, USA). Internal standard (^45^Sc, ^103^Rh, ^125^Te, ^193^Ir) was added via a T-junction in-line (1:20 dilution). Analysis proceeded if oxides and doubly-charged ions were less than 2%. ICP-MS operating parameters are summarised in Supplementary Information S1. Data acquisition and processing were carried out by using MassHunter Workstation (version 4.5). The following isotopes were analysed using He mode: ^11^B, ^23^Na, ^24^ Mg, ^27^Al, ^39^ K, ^44^Ca, ^53^Cr, ^55^Mn, ^56^Fe, ^59^Co, ^60^Ni, ^65^Cu, ^66^Zn, ^71^ Ga, ^85^Rb, ^88^Sr, ^111^Cd, ^133^Cs, ^137^Ba, ^201^Hg,^205^Tl, ^206^Pb, ^207^Pb, ^208^Pb.

### Quality Assurance

Each batch of samples contained method blanks, method filter blanks, duplicates, in-house quality control honey sample, certified reference material ERM-CE278k (mussel tissue) and spiked samples (final concentrations of 8.33 and 83.3 µg^−1^ respectively). During ICP-MS analysis, check standards were analysed every 20 samples and re-calibration was performed every 100 samples. Rinse blank samples were analysed every 10 samples to ensure minimal carryover between samples. SLRS-6 river water CRM was analysed with every batch of samples to check instrument performance.

### Statistical Analysis

Microsoft Excel 2016 and Minitab®21 were used for statistical analyses. For statistical calculation, elements that were not detected (ND) or were < LoD were treated as a missing value for summary statistics (e.g. average); for 2-sample t-test, ANOVA calculations, linear discriminant analysis (with cross-validation), principal component analysis and decision trees they were treated as 0 µg kg^−1^.

## Results and Discussion

### Summary of Elements in Honey

A total of 352 honey samples from 34 countries worldwide were analysed for 20 major, minor and trace elements. A statistical summary of elemental concentrations for all honey samples is presented in Table 1; a summary of elemental concentrations for each country can be found in Supplementary Information S2.

**Table 1:** Summary statistics for elemental concentrations of honey samples collected from 34 countries (*n* = 352)

	*n*	LOD	LOQ	Average	Standard Deviation	Coefficient of variation	Minimum	Q1	Median	Q3	Maximum
		mg kg^−1^									
Na	352	0.100	0.305	63.3	67.8	0.107	8.33	22.5	41.7	77	526
Mg	352	0.100	0.305	31.6	20	0.0634	5.59	19.2	27.5	39.6	215
Al	272	0.100	0.305	5.78	9.22	0.16	0.323	1.13	3.26	8.08	110
K	352	5.03	15.25	1230	664	0.0542	84.5	799	1170	1590	4780
Ca	352	1.00	3.05	61.1	39.7	0.0651	15.9	40.6	49.9	65.5	399
		µg kg^−1^									
B	351	0.100	0.305	5380	2670	49.6	620	3800	4530	6010	16,900
Cr	259	0.100	0.305	23.2	22.3	96.1	8.87	12.8	16.5	24.6	258
Mn	352	0.100	0.305	4350	5140	118	78	778	2280	5900	33,400
Fe	326	50	152.5	1470	1690	115	56	681	1120	1940	25,200
Co	103	0.100	0.305	10.4	13.9	134	1.24	4.8	7.32	11.5	132
Ni	332	0.100	0.305	44.2	115	260	0.15	11.7	19.3	39.3	1200
Cu	351	0.100	0.305	412	333	81	7	167	329	576	2140
Zn	352	10.0	30.5	1550	9140	591	125	573	882	1210	172,000
Rb	352	0.100	0.305	3240	2480	76.6	95	1420	2970	4580	20,600
Sr	352	0.100	0.305	277	1360	492	25.7	110	152	247	25,400
Cd	50	0.100	0.305	12.2	13.9	114	1.45	2.19	6.53	17.6	51.6
Cs	215	0.100	0.305	34.1	48.5	142	0.67	13.7	21.7	37.5	496
Ba	350	0.100	0.305	209	778	372	10	86	136	202	14,400
Tl	93	0.100	0.305	37.2	66.4	178	3.98	9.52	15.3	42.7	537
Pb	110	0.100	0.305	23.3	57.7	248	2.39	10	13	18.8	582

Potassium was the most abundant element found in honey and was present in all samples. It had a mean concentration of 1230 ± 660 mg kg^−1^ and made up approximately 87% of the elemental composition. The next most abundant elements were Na, Ca and Mg, making up 4.49, 4.34 and 2.24% of the total elemental composition of the honey samples, respectively; these four elements were detected in all samples and are readily reported as the most abundant in honey [10, 20, 22, 34]. Elements making up 0.10 – 0.41% of the total elemental profile were (in decreasing order) Al, B, Mn, Rb, Zn and Fe; these elements were present in all samples, apart from B, which was absent from one sample originating from China, and Al and Fe which were present in 77 and 92% of samples respectively. A previous study also noted the low abundance of Al in honey from around the world (present in only 46%, *n* = 69) [30]. Cu, Sr and Ba contributed to 0.01 – 0.03% of the total elemental profile and were detected in all samples, excluding samples from Niue (*n* = 2) that did not contain Ba. The remaining seven elements (Cd, Co, Cr, Cs, Ni, Pb, Tl) made up 0.003% or less of the total elemental concentration and were found in less than 75% of samples (apart from Ni, which was present in 95% of samples); these lower instances are likely to be due to the nature of the elements which are more readily associated with localised or anthropogenic contamination. Of interest, one sample originating from Perth, Australia contained elevated concentrations of Zn and Pb (171 and 0.58 mg kg^−1^respectively); although the hive location is unknown, it is suspected to be near a mining area, which fits with other analyses of Australian honey [35]. In comparison, the next highest concentration of Zn was over a factor of 10 lower (10.4 mg kg^−1^) and all other samples had less than 0.15 mg kg^−1^ Pb. Additionally, one sample from Chile contained elevated Ba and Sr (14.4 mg kg^−1^ and 25.4 mg kg^−1^, respectively); the next highest sample only contained 1.7 mg kg^−1^ Ba and 3.13 mg kg^−1^ Sr (also from Chile). Analysis of a larger number of samples from Chile would be required to determine if honey from this area typically has elevated concentrations of Ba and Sr.

An interesting observation was the presence of Tl in 37% of samples originating from NZ (37.8 ± 67 µg kg^−1^), whereas it was only observed in two international samples (Poland, 14.8 µg kg^−1^ and Chile 10.04 µg kg^−1^). However, the cause of its presence in NZ samples is unknown.

Samples were analysed to determine if there was a difference in concentrations between New Zealand honey samples (*n* = 245) and international samples (*n* = 107) for each element. No statistically significant differences (*p* < 0.05) were observed for Mg, Al, Cr, Fe, Co, Ni, Zn, Sr, Cd, Ba and Pb. For elements that showed a difference in concentration between honey of New Zealand and international origin, B, Na, Mn, Cu, Rb, Cs and Tl each had a p-value < 0.001; K and Ca had p-values of 0.038 and 0.001 respectively.

The full dataset contains samples from 34 countries, however, for some of these countries, less than five samples were analysed. Therefore we also considered a reduced data set that contained countries represented by at least five samples (*n* = 306, 7 countries – NZ, Germany, Denmark, UK, Italy, Greece, Poland). For the subset data, pairwise 2-sample t-tests were conducted to determine if there was a difference between NZ and international samples for any elements; no statistical differences (*p* < 0.05) were observed for Na, Mg, K, Ca, Cr, Fe, Co, Ni, Zn, Cd and Pb. Of the elements that showed a difference in concentration between honey of New Zealand and international origin, B, Al, Mn, Cu, Cs, Ba and Tl each had a p-value < 0.001; Rb and Sr had p-values of 0.001 and 0.023 respectively.

For each element, a one-way analysis of variance (ANOVA) was carried out (*p* < 0.05) to explore differences in elements between all countries. No differences were observed for concentrations of Ca, Co and Pb between any of the countries, suggesting that these three elements are not useful for geographical fingerprinting for this dataset. For Al, Cd, Cs and Tl statistical comparisons could not be completed as at least two countries did not have detectable concentrations of these elements; for example, Al is not present in samples originating from Italy and UK and was only detected in one of five samples from Poland. However, it should be noted that the absence of an element in a country may be useful for discrimination when using multivariate techniques. For authentication of mānuka honey specifically, it was of interest to determine if any elements were statistically different between NZ honey and at least one other country; in addition to Ca, Co and Pb, there was no difference in elemental concentration of Mg and Zn for honey originating from New Zealand when compared with other countries. Elemental concentrations that were different between New Zealand and at least one other country (with the country of difference in parenthesis) were: B and Fe (Germany), Na (all except Italy), K (UK), Cr (all), Mn (Italy, Denmark, Germany, Greece), Ni (Denmark); Cu and Sr (UK, Germany), Rb (Denmark, UK, Italy), Ba (all expect Poland) and Al (Denmark, Germany). It should be noted that samples from Italy and UK did not contain Al, hence were excluded from the analysis, but Al can be included when carrying out multivariate analysis.

These results suggest that some elements (such as B and Cr) may be universally useful for fingerprinting geographical origin of honey, while other elements (e.g., Fe, Mn, Ni, Cu, Sr) may be suitable for discrimination between certain countries.

### Discrimination of New Zealand Honey from International Honey

New Zealand is an isolated country at the bottom of the Pacific Ocean, with Australia as the closest significant landmass. This isolation may allow New Zealand honeys to be discriminated from other countries based on their elemental fingerprint. This work examined honey of non-determined floral origin from New Zealand and international countries.

#### Principal Component Analysis

**Fig. 1:**
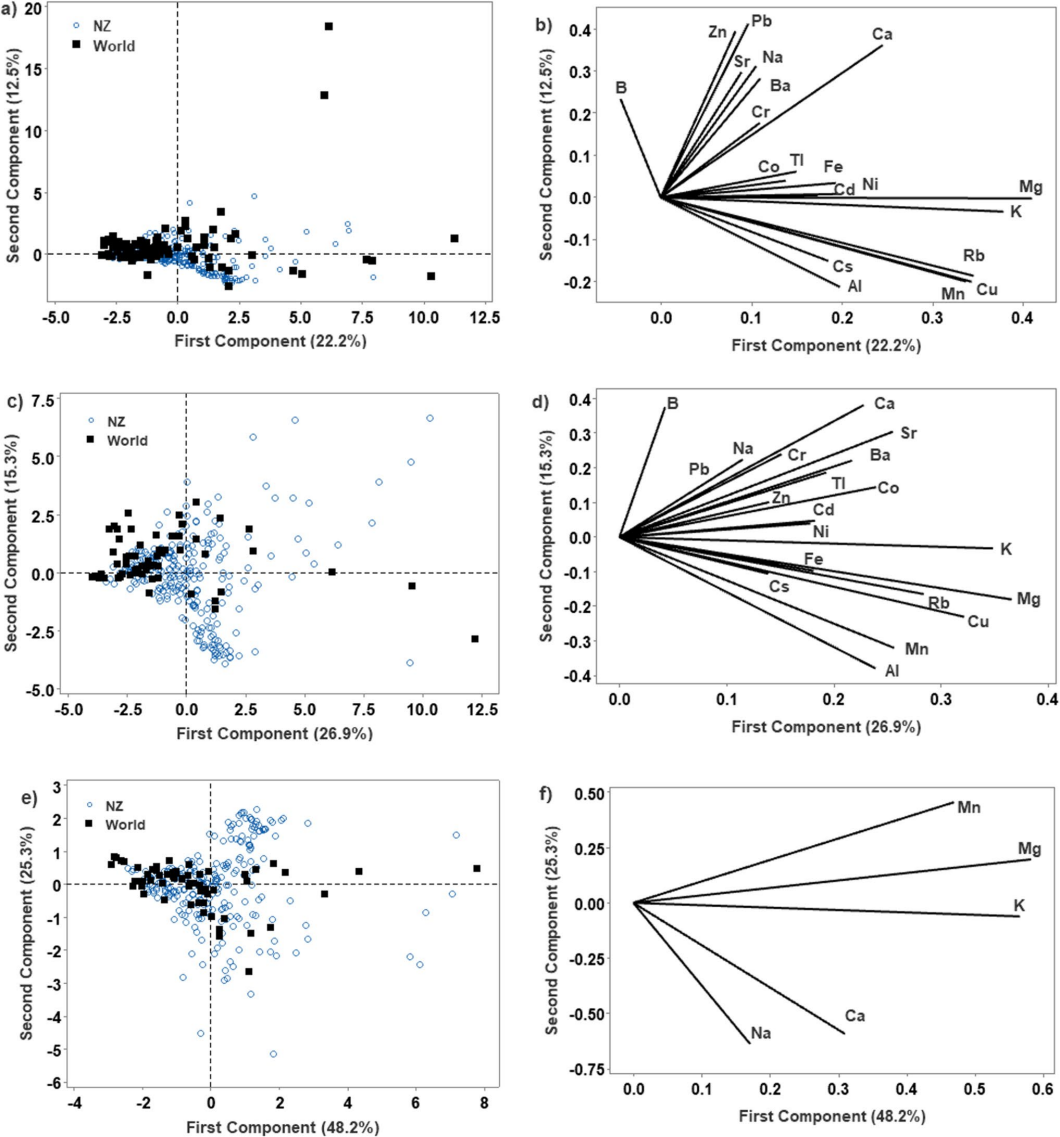
**a** Score plot for PC 1 and 2 using all elements for all samples (*n* = 352); **b** corresponding loading plot for all elements; **c** score plot for PC 1 and 2 using all elements for Europe and NZ subset (*n* = 306); **d** corresponding loading plot for all elements; **e** score plot for PC1 and 2 using soil elements (Ca, K, Mg, Na, Mn) for Europe and NZ subset (*n* = 306); **f** corresponding loading plot for all elements

Firstly, untargeted analysis using principal components analysis (PCA) was carried out to observe groupings based on elemental profile. Analysis of all 20 elements in all samples (*n* = 352) showed that only 34.7% of the data were explained in the first two principal components (PC, 22.2% and 12.5% respectively). The first PC was strongly associated with Mg, K, Mn, Cu and Rb, which all had coefficient values larger than 0.3 in the first PC, while the second PC was associated with Na, Ca, Zn and Pb (coefficient values > 0.3). The two categories did not show visually distinct groupings on the score plot for the first 2 PCs (Fig. 1a, b). Twelve PCs were required to explain 90.2% of the data.

The full dataset contains samples from 34 countries, however, a number of these countries had less than five samples. Therefore the dataset was reduced to contain only countries with at least five samples (*n* = 306, 7 countries); the six non-New Zealand countries were European, hence this subset of data will be referred to as “Europe”.

For the New Zealand and Europe subset of data, the first two PCs explained 42.2% of the data (26.9 and 15.3% respectively, Fig. 1c, d) and 12 PCs were required to explain 91.0% of the data. In the first PC, Mg, K and Cu coefficient values were larger than 0.3. In the second PC, B, Ca and Sr were positively correlated (> 0.3), while Al and Mn were negatively correlated (< –0.3).

In previous work [14], elements associated with soil (Ca, K, Mg, Na and Mn) were used to successfully explain 75.2% of North Island, New Zealand honey samples (*n* = 181). Selecting only elements that are largely influence by the soil removes any variation from point-source contamination. Using these five elements, 73.6% of data was explained in the first two PCs (48.2% and 25.3% respectively); however, the score plot of PC1 and 2 did not show a visual separation of NZ honey compared to honey of European origin (Fig. 1e, f). For this set of data, Ca, Mg, K and Mn were positively correlated with PC 1 (> 0.31); while Mg was positively correlated with PC2 (0.453) and Na and Ca were negatively correlated (> − 0.59).

#### Linear Discriminant Analysis

Linear discriminant analysis (LDA) is a supervised-learning statistical tool that can categorise samples based on their differences. Initially, all international honey was placed into one group to determination if honey originating from New Zealand could be separated from all other countries regardless of origin using all 20 elements. Overall accuracy as either New Zealand or international was 91.5% using all elements (91.8 and 90.7% respectively), using cross-validation. However, due to the low instances of samples for some countries, categorisation into individual countries was not attempted.

LDA was used to determine categorisation accuracy for the subset of New Zealand and Europe samples. Categorisation of this set of samples into either New Zealand or Europe had high overall accuracy (88.9%), with 89.8% of New Zealand samples and 85.2% of Europe samples accurately classified. These results show the potential of elemental analysis as an important tool for identifying New Zealand honey from honey originating from other countries. Categorisation of samples into individual country of origin was also attempted for the NZ/Europe subset (*n* = 306, New Zealand, Denmark, Germany, Greece, Italy, Poland and UK); the overall accuracy was 79.7%; Denmark and New Zealand categories had the highest number of samples correctly assigned (93.3 and 86.5% respectively). Incorrect classifications for New Zealand were placed in Denmark (8), Germany (9), Italy (5), Poland (8) and UK (3).

ICP-MS allows simultaneous detection of many elements on the periodic table, however, in some cases, such as for discrimination and categorisation, using more elements may not afford better data and may hinder the analyses. Elements that alter based on changing external factors, such as localised point source contamination, will have little use for building an elemental fingerprint for certain geographical origins (region or country). For geographical determination, external influences on the selected elements should be considered. For example, Pb may be present due to legacy Pb in petrol or from proximity to mining areas, [35] while Cd may reflect agricultural practices [14] within the flight range of a hive. Some elements, such as Cu and Zn, are present due to natural sources (e.g., soil) but can be elevated by anthropogenic sources. For example, zinc is a component of the Earth’s crust, but anthropogenic activity, such as mining and use in fertilisers and wood preservatives can elevate its concentration in specific locations. With a large sample set, the discrimination model should be able to account for this and put more weight on elements of more importance. However, the exercise of determining the usefulness of certain elements was undertaken by carrying out LDA with reduced elemental sets. Initially, using only elements associated with soil (Ca, K, Mg, Na and Mn), the accuracy decreased to 80.7% (80.4%, NZ; 82.0% international), suggesting that other elements can be helpful for discrimination.

The elemental suite was also refined to exclude elements that were present in less than 70% of samples (percentage of presence shown in parentheses); Cs (61%), Pb (31%), Co (29%), Tl (26%) and Cd (14%). Zhou et al. [30] also used this approach and removed elements from analysis that were in less than 71% of samples. Using the remaining 15 elements, 82.7% of samples were assigned to the correct country of origin, with at least 78% accuracy for UK, NZ, Greece, and Denmark. For determination of whether a honey originates from New Zealand or Europe, 92.5% of data was correctly classified (92.7 and 91.8% respectively), which is slightly higher than the use of all 20 elements. Of the five elements removed, Cd and Pb are likely present due to anthropogenic activity, however, the other elements may be useful for helping categorise by country since New Zealand tends to have a higher abundance of Co and Tl than honey from the other countries analysed. With Cd and Pb removed, the overall accuracy increased to 91.2% (New Zealand, 91.4%; Europe, 90.2%), indicating that elements predominately influenced by point source contamination effect the success of categorisation.

Aceto [13] reported that elements in low abundance from each metal group (i.e. alkali, alkaline earth and transition) are best suited for determining geographical origin. To investigate if refining the selection of elements based on this approach increased discrimination, the following eight elements were chosen: Rb from the alkali metals (excluding Na and K for high abundance and Cs for low instances); Sr and Ba from the alkaline earth metals (excluding Ca and Mg for high abundance); Fe, Cu, Ni and Zn from the first row transitional metals (excluding Mn for high abundance and Co for low instances); Al and B from group 3B were also included. With this set of elements, the accuracy decreased slightly to 89.5% (New Zealand, 90.6%; International, 85.2). This decrease in accuracy may be due to the removal of elements identified as statistically significant between New Zealand and Europe samples (i.e., Ca, Na, K, Ca, Mn). Zhou et al. [30] stated that Ca, Mn, P, K and Sr were the best elements to discriminate Australian honey from overseas honey; this aligns with the current research showing that major elements are required for discrimination at the international level.

If the restraint of requiring an element to be present in > 70% of the database is removed, the elements Cs, Co and Tl can be added into the model (Cd and Pb are excluded due to anthropogenic sources) and the accuracy is 90.2% overall (91% New Zealand, 86.9% Europe respectively). This suggests that the cause of low instances should be taken into consideration. In this case, Cs, Co and Tl are not due to point source contamination but reflective of their geographical origin.

These results show that the statistical analysis is robust with a large set of elements, and it may be beneficial to have a larger number of elements for the model to categorise samples.

#### Decision Tree Analysis

The statistical analyses above have shown elemental analysis to be a promising tool to confirm if a honey is of New Zealand or international origin. An alternative approach for classification is decision tree analysis, which is easy to carry out and interpret. Elements that were below LoD were converted to zero for this analysis. When all samples (*n* = 352) and all elements (*n* = 20) were used to determine whether a sample was of New Zealand or international origin, a decision tree with five terminal nodes (using Cs, Cr, Ni, Tl) gave an optimal tree with a misclassification cost of 0.2031. This successfully assigned 90.6% of samples (91.8 and 87.9% for New Zealand and international respectively). Terminal nodes are chosen based on the misclassification cost and is a balance between the number of nodes required and the level of accuracy. Elemental analysis is often carried out by ICP-MS which is a multi-elemental technique and can simultaneously detect a large number of elements on the periodic table without compromising data integrity. Therefore there is no requirement to limit the number of terminal nodes. For this dataset, the optimal misclassification occurred when using six nodes (0.1611 relative misclassification cost, Supplementary Information S3). The overall accuracy was 92.4% (93.1 and 90.8% accuracy for NZ and international samples respectively). Cs, Ba and Rb were the most important elements for classification (> 82%), whereas Cd, Pb, Sr and Fe were the least useful elements (< 15%). The lowest misclassification occurred when using nine nodes (0.1885 relative misclassification cost); this marginally increased overall accuracy to 29.9% and increased the positive classification of international samples to 92.7% but did not alter correct instances of samples of NZ origin.

Using elements that were present in > 70% of samples (as used in LDA analysis), a 5-node tree was produced, but the overall accuracy was lowered to 86.4% (84.4% and 87.3% for NZ and international respectively; 0.2825 relative misclassification cost). With the removal of Cs from the dataset, Al became the most important element for classification. A 15-node tree had the lowest misclassification cost (0.2509) and increased the overall performance of the model to 89%, however, this is not as effective as the model containing all 20 elements.

The New Zealand/Europe subset of data was assessed; 91.5% of data was correctly classified as NZ (91.8%) or Europe (90.2%) using all 20 elements, 8 nodes and the lowest misclassification cost (0.2046, Supplementary Information S4). The most important elements for classification were Ba, Al and Rb, (> 87%), followed by Cs and Na (77.3 and 76.0% respectively). Aceto [13] noted that Rb was useful in food identification. Cd, Sr, Pb and Co were the least important elements (3.6 – 11.8%). When using elements that were in > 70% of samples or when only removing Cd and Pb from the data set, the accuracy did not increase. A lack of increase in accuracy after reducing the elemental set is due to the ability of the test to determine the elements of best choice. Therefore it is suggested that a large range of elements are analysed by ICP-MS and used for classification analysis for determining geographical origin of honey samples.

### Discrimination and Categorisation of Honey of New Zealand and Denmark/Germany Subset

The dataset includes honey from 34 countries around the world, however, sample numbers are low for most of these countries, which may affect the discriminating power due to natural variation within a country. Aside from New Zealand (*n* = 245), Germany and Denmark had the largest sample sizes (*n* = 20 and 15 respectively). Due to the proximity of Denmark and Germany, an investigation into the similarity of their elemental concentrations was carried out to decide if samples could be pooled. A 2-sample t-test (*p* < 0.05) was carried out; of the 20 elements investigated, no statistical difference was found for Mg, K, Ca, Co, Zn, Rb, Sr, Ba and Pb. Neither country had detectable levels of Cd or Tl; additionally, Denmark did not have detectable Cs. The remaining eight elements (B, Na, Al, Cr, Mn, Fe, Ni and Cu) had statistically different concentrations between Denmark and Germany.

Samples from Denmark and Germany were combined (*n* = 35, Denmark/Germany subset) and the data were examined to observe if there were significant differences in elemental concentrations with New Zealand samples. As expected, the concentrations of K and Ca were not statistically different. Additionally, Cr, Co, Ni and Pb were not statistically significant between the two categories; for Co and Pb this is most likely due to low instances. Eleven elements (B, Na, Mg, Al, Mn, Cu, Zn, Rb, Sr, Cs and Ba had statistically different concentrations (*p* < 0.001) between the two categories. There were not enough instances of Cd and Tl for analysis.

**Fig. 2:**
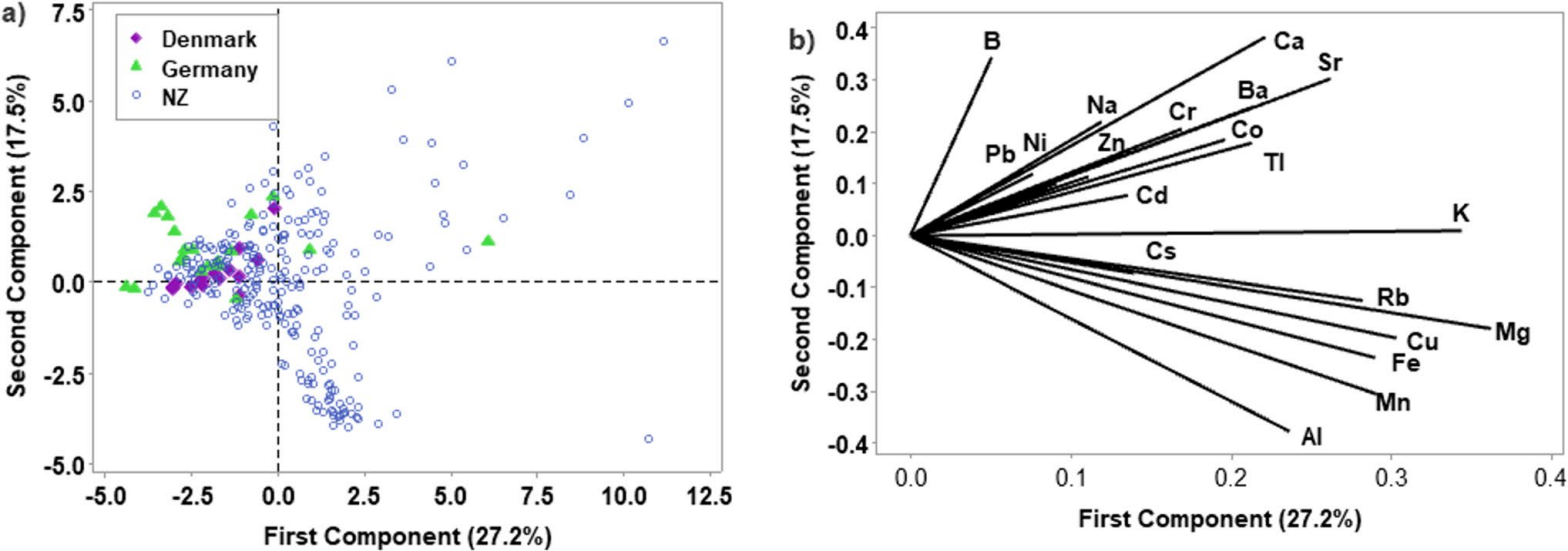
**a** Score plot for PC1 and 2 using all elements for New Zealand, Denmark and Germany samples (*n* = 280); **b** corresponding loading plot for all elements

PCA was carried out using all 20 elements and explained 44.7% of data within the first 2 PCs (27.2 and 17.5% for PC 1 and 2 respectively). It required 11 PCs to explain 90.5% of the variation (Fig. 2 a). The first PC was strongly associated with Mg, K and Cu, which all had coefficient values larger than 0.3 for PC1, while for PC2, B, Ca and Sr had values larger than 0.3, while Al and Mn were more negative than − 0.3 (Fig. 2b). The score plot of PCs 1 and 2 did not show distinct grouping for samples in each category.

Using LDA to examine New Zealand samples compared to the Denmark/Germany subset of samples, an overall accuracy of 91.4% was achieved using all 20 elements and cross-validation, with 91.4% of samples correctly classified in each category.

**Fig. 3:**
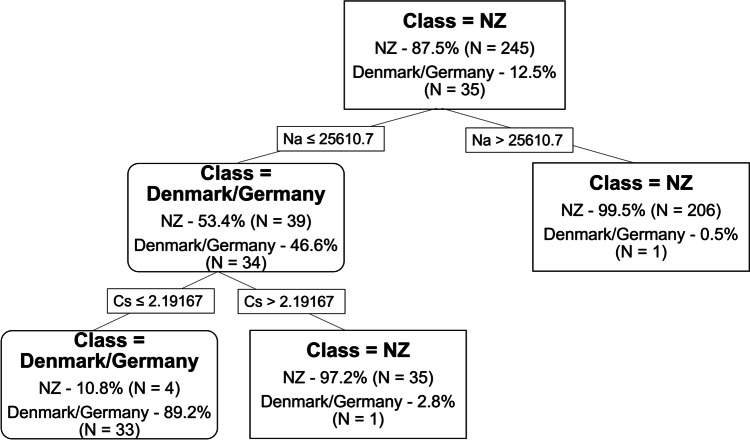
Decision tree with 3 nodes for all NZ and Denmark/Germany samples (*n* = 280) to produce the lowest misclassification cost (0.1469)

Using decision tree analysis with all 20 elements, the ability to classify New Zealand honey from honey originating from the Denmark/Germany subset was possible using three nodes. This tree only only Na and Cs to give the lowest misclassification rate (0.1469, Fig. 3) and correctly assigned 95.7% of samples (96.7 and 88.6% for NZ and Europe subset respectively). Cs (100%) and Rb (95.2%) were the most important elements, followed by Na (82.6%) and Al (81.3%). Using elements that were present in > 70% of samples (as used for LDA analysis), a 3-node tree was produced, but the overall accuracy was marginally lowered to 93.9% (94.7% and 88.6% for NZ and Denmark/Germany subset respectively; 0.1673 relative misclassification cost). With the removal of Cs from the dataset, Na became the second most important element for classification.

## Conclusion

This study shows that the use of elemental profile is a promising tool for discriminating New Zealand honey from international honey and could be used alongside other analyses to confirm honey of New Zealand origin. The various statistical methods explored (PCA, LDA, decision tree) show good agreeance for their ability to categorise honey of New Zealand origin from honey originating from Europe, adding confidence to the ability of elemental analysis. Additionally, the statistical analyses could classify honey as either NZ or Denmark/Germany origin with high accuracy. A larger dataset of honeys from the countries examined in this research and honey from other international countries is required to capture the variation of the honey elemental profile across the world and increase the accuracy of the test. However, this research shows the potential of using elemental fingerprinting for distinguishing honey from NZ origin from different countries.

### Supplementary Information

Below is the link to the electronic supplementary material.

Supplementary file1 (DOCX 14 KB)

Supplementary file2 (DOCX 95 KB)

Supplementary file3 (DOCX 265 KB)

Supplementary file4 (DOCX 390 KB)

## Data Availability

The dataset that are analysed during the study are available from the corresponding author on reasonable request.

## References

[1] Zhao J, Li A, Jin X, Pan L (2020). Technologies in individual animal identification and meat products traceability. Biotechnol Biotechnol Equip.

[2] Ministry for Primary Industries (2021) Apiculture monitoring data. [Online]. Available: https://www.mpi.govt.nz/resources-and-forms/economic-intelligence/farm-monitoring/#apiculture. Accessed 4 Nov 2022

[3] Joint FAO/WHO Codex Alimentarius Commission (2019) CODEX standard for honey CXS 12-1891 *Codex Alimentarius*. Rome: Food and Agriculture Organization of the United Nations: World Health Organization http://www.codexalimentarius.net/search/advanced.do?lang=en

[4] da Silva PM, Gauche C, Gonzaga LV, Costa ACO, Fett R (2016). Honey: Chemical composition, stability and authenticity. Food Chem.

[5] Oroian M, Sonia S, Amariei A, Leahu A, Gutt G (2015). Multi-element composition of honey as a suitable tool for its authenticity analysis. Pol J Food Nutr Sci.

[6] Pisani A, Protano G, Riccobono F (2008). Minor and trace elements in different honey types produced in Siena County (Italy). Food Chem.

[7] Fernández-Torres R, Pérez-Bernal JL, Bello-López MÁ, Callejón-Mochón M, Jiménez-Sánchez JC, Guiraúm-Pérez A (2005). Mineral content and botanical origin of Spanish honeys. Talanta.

[8] Voyslavov T, Mladenova E, Balkanska R (2021). A new approach for determination of the botanical origin of monofloral bee honey, combining mineral content, physicochemical parameters, and self-organizing maps. Molecules.

[9] Jovetić M, Trifković J, Stanković D, Manojlović D, Milojković-Opsenica D (2019). Mineral content as a tool for the assessment of honey authenticity. J AOAC Int.

[10] Uršulin-Trstenjak N, Levanić D, Primorac L, Bošnir J, Vahčić N, Šarić G (2015) Mineral profile of Croatian honey and differences due to its geographical origin. Czech J Food Sci 33(2):156–164. 10.17221/502/2014-CJFS

[11] Nguyen-Quang T, Bui-Quang M, Truong-Ngoc M (2021). Rapid identification of geographical origin of commercial soybean marketed in Vietnam by ICP-MS. J Anal Methods Chem.

[12] Drivelos SA, Georgiou CA (2012). Multi-element and multi-isotope-ratio analysis to determine the geographical origin of foods in the European Union. TrAC Trends Anal Chem.

[13] Aceto M (2016) The use of ICP-MS in food traceability. In: Espiñeira M, Santaclara FJ (eds) Advances in food traceability techniques and technologies. Woodhead Publishing, Sawston, pp. 137–164

[14] Grainger M, Klaus H, Hewitt N, French A (2021). Investigation of inorganic elemental content of honey from regions of North Island, New Zealand. Food Chemistry.

[15] Meister A, Gutierrez-Gines MJ, Maxfield A, Gaw S (2021). Chemical elements and the quality of mānuka (L*eptospermum **scoparium*) honey. Foods.

[16] Czipa N, Diósi G, Phillips C, Kovács B (2017). Examination of honeys and flowers as soil element indicators. Environ Monit Assess.

[17] Gonzalez Paramas AM, Gomez Barez JA, Garcia-Villanova RJ, Rivas Pala T, ArdanuyAlbajar R, Sanchez Sanchez J (2000). Geographical discrimination of honeys by using mineral composition and common chemical quality parameters. J Sci Food Agric.

[18] Atanassova J, Pavlova D, Lazarova M, Yurukova L (2016). Characteristics of honey from serpentine area in the Eastern Rhodopes Mt. Bulgaria. Biol Trace Elem Res.

[19] Caroli S, Forte G, Iamiceli AL, Galoppi B (1999). Determination of essential and potentially toxic trace elements in honey by inductively coupled plasma-based techniques. Talanta.

[20] Di Bella G, Licata P, Potortì AG, Crupi R et al (2020) Mineral content and physico-chemical parameters of honey from North regions of Algeria. Nat Prod Res 1–8. 10.1080/14786419.2020.1791110 10.1080/14786419.2020.1791110 32643412

[21] Di Bella G, Lo Turco V, Potortì AG, Bua GD, Fede MR, Dugo G (2015). Geographical discrimination of Italian honey by multi-element analysis with a chemometric approach. J Food Compost Anal.

[22] Yücel Y, Sultanoğlu P (2013). Characterization of Hatay honeys according to their multi-element analysis using ICP-OES combined with chemometrics. Food Chem.

[23] Karabagias IK, Louppis AP, Kontakos S, Papastephanou C, Kontominas MG (2017). Characterization and geographical discrimination of Greek pine and thyme honeys based on their mineral content, using chemometrics. Eur Food Res Technol.

[24] Fiamegos Y, Dumitrascu C, Ghidotti M, de la CalleGuntiñas MB (2020). Use of energy-dispersive X-ray fluorescence combined with chemometric modelling to classify honey according to botanical variety and geographical origin. Anal Bioanal Chem.

[25] Ghidotti M, Fiamegos Y, Dumitrascu C, de la Calle MB (2021) Use of elemental profiles to verify geographical origin and botanical variety of Spanish honeys with a protected denomination of origin. Food Chemistry 342:128350. 10.1016/j.foodchem.2020.128350 10.1016/j.foodchem.2020.128350 PMC7930469 33092922

[26] Magdas DA, Guyon F, Puscas R, Vigouroux A et al (2021) Applications of emerging stable isotopes and elemental markers for geographical and varietal recognition of Romanian and French honeys. Food Chem 334:127599. 10.1016/j.foodchem.2020.127599 10.1016/j.foodchem.2020.127599 32711278

[27] Baroni MV, Podio NS, Badini RG, Inga M (2015). Linking soil, water, and honey composition to assess the geographical origin of Argentinean honey by multielemental and isotopic analyses. J Agric Food Chem.

[28] Bilandžić N, Sedak M, Đokić M, Bošković AG et al (2019) Element content in ten Croatian honey types from different geographical regions during three seasons. J Food Compost Anal 84:103305. 10.1016/j.jfca.2019.103305

[29] Fechner DC, Hidalgo MJ, Ruiz Díaz JD, Gil RA, Pellerano RG (2020) Geographical origin authentication of honey produced in Argentina. Food Biosci 33:100483. 10.1016/j.fbio.2019.100483

[30] Zhou X, Taylor MP, Salouros H, Prasad S (2018). Authenticity and geographic origin of global honeys determined using carbon isotope ratios and trace elements. Sci Rep.

[31] Danezis GP, Georgiou CA (2022) Elemental metabolomics: Food elemental assessment could reveal geographical origin. Curr Opin Food Sci 100812. 10.1016/j.cofs.2022.100812

[32] Perna AM, Grassi G, Gambacorta E, Simonetti A (2021). Minerals content in Basilicata region (southern Italy) honeys from areas with different anthropic impact. Int J Food Sci Technol.

[33] Grainger M, Hewitt N, French A (2020) Optimised approach for small mass sample preparation and elemental analysis of bees and bee products by inductively coupled plasma mass spectrometry. Talanta 214:120858. 10.1016/j.talanta.2020.120858 10.1016/j.talanta.2020.120858 32278432

[34] Chen H, Fan C, Chang Q, Pang G (2014). Chemometric determination of the botanical origin for Chinese honeys on the basis of mineral elements determined by ICP-MS. J Agric Food Chem.

[35] Zhou X, Taylor MP, Davies PJ, Prasad S (2018) Identifying sources of environmental contamination in European honey bees (*Apis mellifera*) using trace elements and lead isotopic compositions. Environ Sci Technol 52(3):991–1001. 10.1021/acs.est.7b04084 10.1021/acs.est.7b04084 29249154

