# Supplementary material for: Graphical Discrimination of New Zealand Honey from International Honey Using Elemental Analysis
Source: Biol Trace Elem Res. 2023 Apr 29;202(2):754–64. doi: 10.1007/s12011-023-03680-6 (PMC10764415; doi:10.1007/s12011-023-03680-6)
Supplement: Supplementary file 1 — Supplementary file1 (DOCX 14 KB) [file 12011_2023_3680_MOESM1_ESM.docx]

**Graphical discrimination of New Zealand honey from international honey using elemental analysis**

Megan N. C. Grainger^a*^, Hannah Klaus^a^ Nyssa Hewitt^a^, Han Gan^b^, Amanda D. French^a^

*^a^ School of Science, University of Waikato, Private Bag 3105, Hamilton 3240, New Zealand*

*^b^ Department of Mathematics, University of Waikato, Private Bag 3105, Hamilton 3240, New Zealand*

*Corresponding author: [megan.grainger@waikato.ac.nz](about:blank); +64 7 837 9621

**Supplementary Table S1 ICP-MS operating parameters**

| Spectrometer | Agilent 7800 |
| --- | --- |
| Forward (reflected) power (watt) | 1570 |
| Ar Gas flows (L min^-1^):  Plasma  Auxiliary  Nebulizer (carrier gas)  Makeup/Dilution gas  Helium (mL min^-1^) | 15  0.90  0.95  0.10  4.0 |
| Sampling Depth (mm) | 8.0 |
| Detector mode | Pulse counting |
| Octoapole Bias (V) | −8.0 |
| Energy discrimination | 5.0 |
| Number of replicates | 3 |
| Sweeps/replicate | 40 |
| Dwell time (s) | 0.01 - 0.1 |
