# Supplementary material for: Graphical Discrimination of New Zealand Honey from International Honey Using Elemental Analysis
Source: Biol Trace Elem Res. 2023 Apr 29;202(2):754–64. doi: 10.1007/s12011-023-03680-6 (PMC10764415; doi:10.1007/s12011-023-03680-6)
Supplement: Supplementary file 2 — Supplementary file2 (DOCX 95 KB) [file 12011_2023_3680_MOESM2_ESM.docx]

**Graphical discrimination of New Zealand honey from international honey using elemental analysis**

Megan N. C. Grainger^a*^, Hannah Klaus^a^ Nyssa Hewitt^a^, Han Gan^b^, Amanda D. French^a^

*^a^ School of Science, University of Waikato, Private Bag 3105, Hamilton 3240, New Zealand*

*^b^ Department of Mathematics, University of Waikato, Private Bag 3105, Hamilton 3240, New Zealand*

*Corresponding author: [megan.grainger@waikato.ac.nz](about:blank); +64 7 837 9621

**Supplementary Table S2 Summary of individual elements categorised by country**

|  | **Country** | **>DL** | **<DL** | **Mean** | **StDev** | **CoefVar** | **Minimum** | **Q1** | **Median** | **Q3** | **Maximum** | **Range** |
| --- | --- | --- | --- | --- | --- | --- | --- | --- | --- | --- | --- | --- |
| **B** | Argentina | 1 | 0 | 9420 | * | * | 9420 | * | 9420 | * | 9420 | * |
|  | Australia | 3 | 0 | 5820 | 1750 | 30 | 4130 | 4130 | 5720 | 7620 | 7620 | 3490 |
|  | Austria | 2 | 0 | 4940 | 583 | 11.8 | 4530 | * | 4940 | * | 5350 | 825 |
|  | Belgium | 1 | 0 | 7440 | * | * | 7440 | * | 7440 | * | 7440 | * |
|  | Belize | 2 | 0 | 7600 | 4420 | 58.2 | 4480 | * | 7600 | * | 10700 | 6250 |
|  | Chile | 3 | 0 | 5510 | 2540 | 46.1 | 3640 | 3640 | 4490 | 8400 | 8400 | 4760 |
|  | China | 1 | 1 | 4520 | * | * | 4520 | * | 4520 | * | 4520 | * |
|  | Croatia | 1 | 0 | 3660 | * | * | 3660 | * | 3660 | * | 3660 | * |
|  | Denmark | 15 | 0 | 5900 | 2040 | 34.5 | 3680 | 4590 | 5520 | 6240 | 11600 | 7960 |
|  | Finland | 1 | 0 | 10700 | * | * | 10700 | * | 10700 | * | 10700 | * |
|  | France | 2 | 0 | 9900 | 5680 | 57.4 | 5880 | * | 9900 | * | 13900 | 8040 |
|  | Georgia | 1 | 0 | 1170 | * | * | 1170 | * | 1170 | * | 1170 | * |
|  | Germany | 20 | 0 | 8710 | 4030 | 46.3 | 3930 | 5190 | 7780 | 12500 | 15500 | 11600 |
|  | Greece | 9 | 0 | 5040 | 3790 | 75.1 | 620 | 2470 | 4430 | 6510 | 13400 | 12800 |
|  | India | 4 | 0 | 4650 | 982 | 21.1 | 3580 | 3760 | 4550 | 5630 | 5920 | 2340 |
|  | Italy | 7 | 0 | 5810 | 977 | 16.8 | 4950 | 5140 | 5500 | 6210 | 7810 | 2860 |
|  | Japan | 1 | 0 | 3750 | * | * | 3750 | * | 3750 | * | 3750 | * |
|  | Madeira | 1 | 0 | 4580 | * | * | 4580 | * | 4580 | * | 4580 | * |
|  | Maledives | 1 | 0 | 2900 | * | * | 2900 | * | 2900 | * | 2900 | * |
|  | Mongolia | 1 | 0 | 2750 | * | * | 2750 | * | 2750 | * | 2750 | * |
|  | Morocco | 1 | 0 | 12600 | * | * | 12600 | * | 12600 | * | 12600 | * |
|  | Niue | 2 | 0 | 5890 | 1340 | 22.8 | 4940 | * | 5890 | * | 6840 | 1900 |
|  | NZ | 245 | 0 | 4810 | 1980 | 41.1 | 2250 | 3640 | 4260 | 5260 | 14100 | 11900 |
|  | Poland | 5 | 0 | 6350 | 2390 | 37.7 | 3230 | 3980 | 6780 | 8510 | 9220 | 5990 |
|  | Portugal | 1 | 0 | 7070 | * | * | 7070 | * | 7070 | * | 7070 | * |
|  | Russia | 4 | 0 | 8200 | 5690 | 69.4 | 4340 | 4610 | 5910 | 14100 | 16600 | 12300 |
|  | S. America | 1 | 0 | 9130 | * | * | 9130 | * | 9130 | * | 9130 | * |
|  | South Africa | 1 | 0 | 2020 | * | * | 2020 | * | 2020 | * | 2020 | * |
|  | Spain | 3 | 0 | 7320 | 1470 | 20.1 | 6000 | 6000 | 7050 | 8910 | 8910 | 2910 |
|  | Turkey | 2 | 0 | 7370 | 1870 | 25.4 | 6050 | * | 7370 | * | 8690 | 2640 |
|  | UK | 5 | 0 | 10000 | 4860 | 48.7 | 5720 | 6140 | 7520 | 15100 | 16900 | 11200 |
|  | USA | 2 | 0 | 6720 | 6020 | 89.5 | 2470 | * | 6720 | * | 11000 | 8520 |
|  | Uzbekistan | 1 | 0 | 5870 | * | * | 5870 | * | 5870 | * | 5870 | * |
|  | Vietnam | 1 | 0 | 650 | * | * | 650 | * | 650 | * | 650 | * |
| **Na** | Argentina | 1 | 0 | 32900 | * | * | 32900 | * | 32900 | * | 32900 | * |
|  | Australia | 3 | 0 | 281000 | 212000 | 75.5 | 158000 | 158000 | 159000 | 526000 | 526000 | 368000 |
|  | Austria | 2 | 0 | 17900 | 3480 | 19.4 | 15400 | * | 17900 | * | 20400 | 4920 |
|  | Belgium | 1 | 0 | 15300 | * | * | 15300 | * | 15300 | * | 15300 | * |
|  | Belize | 2 | 0 | 24600 | 4600 | 18.7 | 21400 | * | 24600 | * | 27900 | 6510 |
|  | Chile | 3 | 0 | 22500 | 10800 | 47.8 | 13400 | 13400 | 19800 | 34400 | 34400 | 21000 |
|  | China | 2 | 0 | 14800 | 720 | 4.87 | 14300 | * | 14800 | * | 15300 | 1020 |
|  | Croatia | 1 | 0 | 17800 | * | * | 17800 | * | 17800 | * | 17800 | * |
|  | Denmark | 15 | 0 | 11100 | 3080 | 27.9 | 8330 | 9010 | 9820 | 12900 | 19200 | 10900 |
|  | Finland | 1 | 0 | 30500 | * | * | 30500 | * | 30500 | * | 30500 | * |
|  | France | 2 | 0 | 17200 | 1780 | 10.4 | 15900 | * | 17200 | * | 18400 | 2510 |
|  | Georgia | 1 | 0 | 25200 | * | * | 25200 | * | 25200 | * | 25200 | * |
|  | Germany | 20 | 0 | 18200 | 5130 | 28.1 | 10300 | 14000 | 17300 | 23200 | 27300 | 17000 |
|  | Greece | 9 | 0 | 32700 | 13400 | 40.9 | 11600 | 22500 | 32300 | 39900 | 58000 | 46400 |
|  | India | 4 | 0 | 85600 | 143000 | 167 | 12700 | 12800 | 15200 | 229000 | 299000 | 287000 |
|  | Italy | 7 | 0 | 54900 | 55800 | 102 | 17700 | 18100 | 29600 | 97600 | 165000 | 147000 |
|  | Japan | 1 | 0 | 23300 | * | * | 23300 | * | 23300 | * | 23300 | * |
|  | Madeira | 1 | 0 | 118000 | * | * | 118000 | * | 118000 | * | 118000 | * |
|  | Maledives | 1 | 0 | 43000 | * | * | 43000 | * | 43000 | * | 43000 | * |
|  | Mongolia | 1 | 0 | 25700 | * | * | 25700 | * | 25700 | * | 25700 | * |
|  | Morocco | 1 | 0 | 24100 | * | * | 24100 | * | 24100 | * | 24100 | * |
|  | Niue | 2 | 0 | 244000 | 91900 | 37.7 | 179000 | * | 244000 | * | 309000 | 130000 |
|  | NZ | 245 | 0 | 73700 | 65100 | 88.3 | 9880 | 31900 | 57400 | 87500 | 489000 | 479000 |
|  | Poland | 5 | 0 | 27400 | 5880 | 21.5 | 21600 | 22400 | 24900 | 33500 | 35200 | 13600 |
|  | Portugal | 1 | 0 | 44600 | * | * | 44600 | * | 44600 | * | 44600 | * |
|  | Russia | 4 | 0 | 11900 | 2410 | 20.2 | 9040 | 9640 | 11900 | 14200 | 14900 | 5820 |
|  | S. America | 1 | 0 | 70500 | * | * | 70500 | * | 70500 | * | 70500 | * |
|  | South Africa | 1 | 0 | 37400 | * | * | 37400 | * | 37400 | * | 37400 | * |
|  | Spain | 3 | 0 | 42200 | 7990 | 18.9 | 34600 | 34600 | 41500 | 50500 | 50500 | 15900 |
|  | Turkey | 2 | 0 | 37600 | 8530 | 22.7 | 31500 | * | 37600 | * | 43600 | 12100 |
|  | UK | 5 | 0 | 23500 | 6960 | 29.7 | 16800 | 18500 | 22500 | 28900 | 35200 | 18400 |
|  | USA | 2 | 0 | 22100 | 8250 | 37.3 | 16300 | * | 22100 | * | 27900 | 11700 |
|  | Uzbekistan | 1 | 0 | 21300 | * | * | 21300 | * | 21300 | * | 21300 | * |
|  | Vietnam | 1 | 0 | 49700 | * | * | 49700 | * | 49700 | * | 49700 | * |
| **Mg** | Argentina | 1 | 0 | 13000 | * | * | 13000 | * | 13000 | * | 13000 | * |
|  | Australia | 3 | 0 | 51200 | 42000 | 82 | 21500 | 21500 | 32900 | 99200 | 99200 | 77700 |
|  | Austria | 2 | 0 | 42600 | 32100 | 75.4 | 19900 | * | 42600 | * | 65300 | 45400 |
|  | Belgium | 1 | 0 | 14300 | * | * | 14300 | * | 14300 | * | 14300 | * |
|  | Belize | 2 | 0 | 16100 | 6210 | 38.6 | 11700 | * | 16100 | * | 20500 | 8780 |
|  | Chile | 3 | 0 | 23400 | 11900 | 50.8 | 12500 | 12500 | 21700 | 36100 | 36100 | 23600 |
|  | China | 2 | 0 | 10100 | 3740 | 37.1 | 7450 | * | 10100 | * | 12700 | 5290 |
|  | Croatia | 1 | 0 | 38200 | * | * | 38200 | * | 38200 | * | 38200 | * |
|  | Denmark | 15 | 0 | 27600 | 7650 | 27.7 | 13000 | 22600 | 27600 | 31700 | 41400 | 28400 |
|  | Finland | 1 | 0 | 26100 | * | * | 26100 | * | 26100 | * | 26100 | * |
|  | France | 2 | 0 | 22000 | 11600 | 53.1 | 13700 | * | 22000 | * | 30200 | 16500 |
|  | Georgia | 1 | 0 | 16500 | * | * | 16500 | * | 16500 | * | 16500 | * |
|  | Germany | 20 | 0 | 21900 | 12900 | 58.9 | 7180 | 14800 | 19300 | 26700 | 66000 | 58800 |
|  | Greece | 9 | 0 | 52700 | 33200 | 63.1 | 32900 | 34300 | 47200 | 52500 | 138000 | 105000 |
|  | India | 4 | 0 | 103000 | 81900 | 79.9 | 32500 | 37400 | 81300 | 189000 | 215000 | 182000 |
|  | Italy | 7 | 0 | 22700 | 19200 | 84.5 | 8010 | 8650 | 17000 | 37300 | 59600 | 51600 |
|  | Japan | 1 | 0 | 13100 | * | * | 13100 | * | 13100 | * | 13100 | * |
|  | Madeira | 1 | 0 | 37100 | * | * | 37100 | * | 37100 | * | 37100 | * |
|  | Maledives | 1 | 0 | 19000 | * | * | 19000 | * | 19000 | * | 19000 | * |
|  | Mongolia | 1 | 0 | 8140 | * | * | 8140 | * | 8140 | * | 8140 | * |
|  | Morocco | 1 | 0 | 7250 | * | * | 7250 | * | 7250 | * | 7250 | * |
|  | Niue | 2 | 0 | 81600 | 16600 | 20.3 | 69900 | * | 81600 | * | 93300 | 23400 |
|  | NZ | 245 | 0 | 31800 | 14600 | 45.8 | 6850 | 21800 | 29200 | 40900 | 102000 | 95600 |
|  | Poland | 5 | 0 | 40100 | 37500 | 93.7 | 5590 | 13300 | 29800 | 72000 | 103000 | 97500 |
|  | Portugal | 1 | 0 | 18700 | * | * | 18700 | * | 18700 | * | 18700 | * |
|  | Russia | 4 | 0 | 14200 | 8040 | 56.7 | 7780 | 8090 | 11800 | 22700 | 25400 | 17600 |
|  | S. America | 1 | 0 | 49800 | * | * | 49800 | * | 49800 | * | 49800 | * |
|  | South Africa | 1 | 0 | 6320 | * | * | 6320 | * | 6320 | * | 6320 | * |
|  | Spain | 3 | 0 | 36200 | 10500 | 29.2 | 28100 | 28100 | 32300 | 48100 | 48100 | 20000 |
|  | Turkey | 2 | 0 | 18300 | 10300 | 56.4 | 11000 | * | 18300 | * | 25600 | 14600 |
|  | UK | 5 | 0 | 20500 | 7850 | 38.3 | 14500 | 15400 | 16500 | 27600 | 33700 | 19300 |
|  | USA | 2 | 0 | 26600 | 20700 | 77.6 | 12000 | * | 26600 | * | 41200 | 29200 |
|  | Uzbekistan | 1 | 0 | 17700 | * | * | 17700 | * | 17700 | * | 17700 | * |
|  | Vietnam | 1 | 0 | 19200 | * | * | 19200 | * | 19200 | * | 19200 | * |
| **Al** | Argentina | 0 | 1 | * | * | * | * | * | * | * | * | * |
|  | Australia | 1 | 2 | 2100 | * | * | 2100 | * | 2100 | * | 2100 | * |
|  | Austria | 1 | 1 | 7000 | * | * | 7000 | * | 7000 | * | 7000 | * |
|  | Belgium | 0 | 1 | * | * | * | * | * | * | * | * | * |
|  | Belize | 0 | 2 | * | * | * | * | * | * | * | * | * |
|  | Chile | 3 | 0 | 2050 | 1130 | 55.3 | 1150 | 1150 | 1670 | 3320 | 3320 | 2170 |
|  | China | 1 | 1 | 64200 | * | * | 64200 | * | 64200 | * | 64200 | * |
|  | Croatia | 0 | 1 | * | * | * | * | * | * | * | * | * |
|  | Denmark | 11 | 4 | 887 | 350 | 39.5 | 636 | 643 | 783 | 1030 | 1830 | 1190 |
|  | Finland | 0 | 1 | * | * | * | * | * | * | * | * | * |
|  | France | 0 | 2 | * | * | * | * | * | * | * | * | * |
|  | Georgia | 1 | 0 | 110000 | * | * | 110000 | * | 110000 | * | 110000 | * |
|  | Germany | 2 | 18 | 1770 | 1240 | 70.2 | 889 | * | 1770 | * | 2640 | 1750 |
|  | Greece | 6 | 3 | 8920 | 14000 | 157 | 843 | 970 | 1810 | 18000 | 36100 | 35300 |
|  | India | 4 | 0 | 8470 | 5510 | 65.1 | 1590 | 3230 | 8610 | 13600 | 15100 | 13500 |
|  | Italy | 0 | 7 | * | * | * | * | * | * | * | * | * |
|  | Japan | 1 | 0 | 848 | * | * | 848 | * | 848 | * | 848 | * |
|  | Madeira | 1 | 0 | 786 | * | * | 786 | * | 786 | * | 786 | * |
|  | Maledives | 0 | 1 | * | * | * | * | * | * | * | * | * |
|  | Mongolia | 0 | 1 | * | * | * | * | * | * | * | * | * |
|  | Morocco | 1 | 0 | 1280 | * | * | 1280 | * | 1280 | * | 1280 | * |
|  | Niue | 1 | 1 | 490 | * | * | 490 | * | 490 | * | 490 | * |
|  | NZ | 232 | 13 | 5400 | 5440 | 101 | 323 | 1320 | 3720 | 8250 | 45300 | 44900 |
|  | Poland | 1 | 4 | 15600 | * | * | 15600 | * | 15600 | * | 15600 | * |
|  | Portugal | 0 | 1 | * | * | * | * | * | * | * | * | * |
|  | Russia | 0 | 4 | * | * | * | * | * | * | * | * | * |
|  | S. America | 1 | 0 | 3250 | * | * | 3250 | * | 3250 | * | 3250 | * |
|  | South Africa | 0 | 1 | * | * | * | * | * | * | * | * | * |
|  | Spain | 1 | 2 | 813 | * | * | 813 | * | 813 | * | 813 | * |
|  | Turkey | 1 | 1 | 1480 | * | * | 1480 | * | 1480 | * | 1480 | * |
|  | UK | 0 | 5 | * | * | * | * | * | * | * | * | * |
|  | USA | 1 | 1 | 1840 | * | * | 1840 | * | 1840 | * | 1840 | * |
|  | Uzbekistan | 0 | 1 | * | * | * | * | * | * | * | * | * |
|  | Vietnam | 1 | 0 | 2480 | * | * | 2480 | * | 2480 | * | 2480 | * |
| **K** | Argentina | 1 | 0 | 392000 | * | * | 392000 | * | 392000 | * | 392000 | * |
|  | Australia | 3 | 0 | 1960000 | 1030000 | 52.6 | 1180000 | 1180000 | 1570000 | 3130000 | 3130000 | 1950000 |
|  | Austria | 2 | 0 | 1430000 | 203000 | 14.2 | 1280000 | * | 1430000 | * | 1570000 | 287000 |
|  | Belgium | 1 | 0 | 654000 | * | * | 654000 | * | 654000 | * | 654000 | * |
|  | Belize | 2 | 0 | 295000 | 217000 | 73.4 | 142000 | * | 295000 | * | 448000 | 306000 |
|  | Chile | 3 | 0 | 1480000 | 691000 | 46.8 | 877000 | 877000 | 1320000 | 2230000 | 2230000 | 1350000 |
|  | China | 2 | 0 | 145000 | 86000 | 59.2 | 84500 | * | 145000 | * | 206000 | 122000 |
|  | Croatia | 1 | 0 | 2950000 | * | * | 2950000 | * | 2950000 | * | 2950000 | * |
|  | Denmark | 15 | 0 | 1120000 | 282000 | 25.2 | 533000 | 940000 | 1090000 | 1380000 | 1690000 | 1160000 |
|  | Finland | 1 | 0 | 569000 | * | * | 569000 | * | 569000 | * | 569000 | * |
|  | France | 2 | 0 | 1180000 | 1410000 | 120 | 182000 | * | 1180000 | * | 2180000 | 2000000 |
|  | Georgia | 1 | 0 | 1130000 | * | * | 1130000 | * | 1130000 | * | 1130000 | * |
|  | Germany | 20 | 0 | 1090000 | 850000 | 78.2 | 131000 | 271000 | 1000000 | 1640000 | 3670000 | 3530000 |
|  | Greece | 9 | 0 | 1910000 | 1390000 | 73 | 722000 | 854000 | 1130000 | 2870000 | 4780000 | 4060000 |
|  | India | 4 | 0 | 1870000 | 1190000 | 63.4 | 595000 | 773000 | 1780000 | 3060000 | 3330000 | 2730000 |
|  | Italy | 7 | 0 | 1000000 | 719000 | 71.8 | 345000 | 369000 | 643000 | 1610000 | 2070000 | 1720000 |
|  | Japan | 1 | 0 | 1260000 | * | * | 1260000 | * | 1260000 | * | 1260000 | * |
|  | Madeira | 1 | 0 | 1670000 | * | * | 1670000 | * | 1670000 | * | 1670000 | * |
|  | Maledives | 1 | 0 | 450000 | * | * | 450000 | * | 450000 | * | 450000 | * |
|  | Mongolia | 1 | 0 | 149000 | * | * | 149000 | * | 149000 | * | 149000 | * |
|  | Morocco | 1 | 0 | 161000 | * | * | 161000 | * | 161000 | * | 161000 | * |
|  | Niue | 2 | 0 | 1640000 | 195000 | 11.9 | 1500000 | * | 1640000 | * | 1780000 | 276000 |
|  | NZ | 245 | 0 | 1280000 | 551000 | 42.9 | 230000 | 874000 | 1240000 | 1610000 | 3610000 | 3380000 |
|  | Poland | 5 | 0 | 1230000 | 964000 | 78.4 | 149000 | 272000 | 1430000 | 2080000 | 2500000 | 2350000 |
|  | Portugal | 1 | 0 | 1270000 | * | * | 1270000 | * | 1270000 | * | 1270000 | * |
|  | Russia | 4 | 0 | 237000 | 80100 | 33.9 | 131000 | 156000 | 246000 | 308000 | 324000 | 193000 |
|  | S. America | 1 | 0 | 845000 | * | * | 845000 | * | 845000 | * | 845000 | * |
|  | South Africa | 1 | 0 | 136000 | * | * | 136000 | * | 136000 | * | 136000 | * |
|  | Spain | 3 | 0 | 1090000 | 380000 | 34.9 | 720000 | 720000 | 1070000 | 1480000 | 1480000 | 760000 |
|  | Turkey | 2 | 0 | 449000 | 350000 | 77.9 | 202000 | * | 449000 | * | 697000 | 495000 |
|  | UK | 5 | 0 | 473000 | 292000 | 61.9 | 224000 | 253000 | 314000 | 772000 | 912000 | 688000 |
|  | USA | 2 | 0 | 578000 | 613000 | 106 | 144000 | * | 578000 | * | 1010000 | 867000 |
|  | Uzbekistan | 1 | 0 | 711000 | * | * | 711000 | * | 711000 | * | 711000 | * |
|  | Vietnam | 1 | 0 | 178000 | * | * | 178000 | * | 178000 | * | 178000 | * |
| **Ca** | Argentina | 1 | 0 | 44300 | * | * | 44300 | * | 44300 | * | 44300 | * |
|  | Australia | 3 | 0 | 171000 | 65700 | 38.5 | 96500 | 96500 | 193000 | 222000 | 222000 | 125000 |
|  | Austria | 2 | 0 | 34800 | 11700 | 33.5 | 26600 | * | 34800 | * | 43100 | 16500 |
|  | Belgium | 1 | 0 | 33800 | * | * | 33800 | * | 33800 | * | 33800 | * |
|  | Belize | 2 | 0 | 46200 | 15000 | 32.4 | 35600 | * | 46200 | * | 56800 | 21200 |
|  | Chile | 3 | 0 | 160000 | 154000 | 96 | 38600 | 38600 | 109000 | 333000 | 333000 | 294000 |
|  | China | 2 | 0 | 38700 | 32200 | 83.2 | 15900 | * | 38700 | * | 61500 | 45500 |
|  | Croatia | 1 | 0 | 158000 | * | * | 158000 | * | 158000 | * | 158000 | * |
|  | Denmark | 15 | 0 | 55600 | 15400 | 27.6 | 34900 | 47200 | 53900 | 60500 | 87500 | 52600 |
|  | Finland | 1 | 0 | 63900 | * | * | 63900 | * | 63900 | * | 63900 | * |
|  | France | 2 | 0 | 116000 | 82700 | 71.5 | 57100 | * | 116000 | * | 174000 | 117000 |
|  | Georgia | 1 | 0 | 116000 | * | * | 116000 | * | 116000 | * | 116000 | * |
|  | Germany | 20 | 0 | 56500 | 23100 | 40.8 | 17800 | 46400 | 53800 | 59100 | 107000 | 88900 |
|  | Greece | 9 | 0 | 88200 | 52000 | 59 | 32100 | 40500 | 93200 | 122000 | 188000 | 155000 |
|  | India | 4 | 0 | 214000 | 155000 | 72.4 | 62700 | 75200 | 197000 | 370000 | 399000 | 336000 |
|  | Italy | 7 | 0 | 52500 | 23300 | 44.3 | 19100 | 21200 | 64700 | 71600 | 71800 | 52700 |
|  | Japan | 1 | 0 | 51700 | * | * | 51700 | * | 51700 | * | 51700 | * |
|  | Madeira | 1 | 0 | 72000 | * | * | 72000 | * | 72000 | * | 72000 | * |
|  | Maledives | 1 | 0 | 70800 | * | * | 70800 | * | 70800 | * | 70800 | * |
|  | Mongolia | 1 | 0 | 26900 | * | * | 26900 | * | 26900 | * | 26900 | * |
|  | Morocco | 1 | 0 | 31400 | * | * | 31400 | * | 31400 | * | 31400 | * |
|  | Niue | 2 | 0 | 107000 | 1170 | 1.1 | 106000 | * | 107000 | * | 108000 | 1660 |
|  | NZ | 245 | 0 | 55100 | 24100 | 43.7 | 23900 | 39900 | 47900 | 59300 | 173000 | 149000 |
|  | Poland | 5 | 0 | 74200 | 44600 | 60.2 | 17300 | 30100 | 82400 | 114000 | 130000 | 112000 |
|  | Portugal | 1 | 0 | 105000 | * | * | 105000 | * | 105000 | * | 105000 | * |
|  | Russia | 4 | 0 | 37300 | 26600 | 71.3 | 16600 | 19200 | 28200 | 64700 | 76400 | 59800 |
|  | S. America | 1 | 0 | 158000 | * | * | 158000 | * | 158000 | * | 158000 | * |
|  | South Africa | 1 | 0 | 19200 | * | * | 19200 | * | 19200 | * | 19200 | * |
|  | Spain | 3 | 0 | 59900 | 25600 | 42.8 | 30300 | 30300 | 74500 | 74900 | 74900 | 44600 |
|  | Turkey | 2 | 0 | 61800 | 1360 | 2.19 | 60800 | * | 61800 | * | 62800 | 1920 |
|  | UK | 5 | 0 | 55100 | 19100 | 34.7 | 34200 | 38000 | 52200 | 73700 | 82400 | 48200 |
|  | USA | 2 | 0 | 52400 | 10100 | 19.3 | 45300 | * | 52400 | * | 59600 | 14300 |
|  | Uzbekistan | 1 | 0 | 47300 | * | * | 47300 | * | 47300 | * | 47300 | * |
|  | Vietnam | 1 | 0 | 128000 | * | * | 128000 | * | 128000 | * | 128000 | * |
| **Cr** | Argentina | 1 | 0 | 13.6 | * | * | 13.6 | * | 13.6 | * | 13.6 | * |
|  | Australia | 3 | 0 | 18.5 | 9.85 | 53.2 | 10.5 | 10.5 | 15.6 | 29.5 | 29.5 | 19 |
|  | Austria | 2 | 0 | 20.2 | 5.22 | 25.8 | 16.5 | * | 20.2 | * | 23.9 | 7.38 |
|  | Belgium | 1 | 0 | 10.1 | * | * | 10.1 | * | 10.1 | * | 10.1 | * |
|  | Belize | 2 | 0 | 30.9 | 25.9 | 83.9 | 12.6 | * | 30.9 | * | 49.3 | 36.7 |
|  | Chile | 2 | 1 | 12.8 | 0.701 | 5.46 | 12.3 | * | 12.8 | * | 13.3 | 0.991 |
|  | China | 2 | 0 | 19.6 | 1.53 | 7.79 | 18.5 | * | 19.6 | * | 20.7 | 2.16 |
|  | Croatia | 1 | 0 | 14.7 | * | * | 14.7 | * | 14.7 | * | 14.7 | * |
|  | Denmark | 15 | 0 | 12.4 | 2.56 | 20.7 | 9.72 | 10.7 | 11.6 | 13.8 | 19.6 | 9.85 |
|  | Finland | 1 | 0 | 15 | * | * | 15 | * | 15 | * | 15 | * |
|  | France | 2 | 0 | 16.2 | 6.12 | 37.9 | 11.8 | * | 16.2 | * | 20.5 | 8.66 |
|  | Georgia | 1 | 0 | 16.3 | * | * | 16.3 | * | 16.3 | * | 16.3 | * |
|  | Germany | 20 | 0 | 16.8 | 5.09 | 30.3 | 10.2 | 13.1 | 15.4 | 18.8 | 30.4 | 20.3 |
|  | Greece | 9 | 0 | 15.2 | 2.19 | 14.4 | 12.3 | 12.9 | 15.2 | 17.1 | 18.4 | 6.19 |
|  | India | 4 | 0 | 19.7 | 7.08 | 36 | 15.1 | 15.3 | 16.7 | 27 | 30.2 | 15 |
|  | Italy | 7 | 0 | 16.1 | 3.07 | 19 | 13.3 | 13.4 | 14.7 | 18.7 | 20.8 | 7.54 |
|  | Japan | 1 | 0 | 13.4 | * | * | 13.4 | * | 13.4 | * | 13.4 | * |
|  | Madeira | 1 | 0 | 25.1 | * | * | 25.1 | * | 25.1 | * | 25.1 | * |
|  | Maledives | 1 | 0 | 36.4 | * | * | 36.4 | * | 36.4 | * | 36.4 | * |
|  | Mongolia | 1 | 0 | 26.7 | * | * | 26.7 | * | 26.7 | * | 26.7 | * |
|  | Morocco | 1 | 0 | 11.8 | * | * | 11.8 | * | 11.8 | * | 11.8 | * |
|  | Niue | 1 | 1 | 17.1 | * | * | 17.1 | * | 17.1 | * | 17.1 | * |
|  | NZ | 155 | 90 | 27.8 | 27.4 | 98.4 | 8.87 | 13.3 | 18.7 | 30.7 | 258 | 250 |
|  | Poland | 5 | 0 | 14.8 | 4.49 | 30.3 | 11.1 | 11.3 | 13.2 | 19.2 | 21.9 | 10.8 |
|  | Portugal | 1 | 0 | 16 | * | * | 16 | * | 16 | * | 16 | * |
|  | Russia | 4 | 0 | 13.8 | 1.88 | 13.6 | 12 | 12.2 | 13.5 | 15.8 | 16.3 | 4.34 |
|  | S. America | 1 | 0 | 22.6 | * | * | 22.6 | * | 22.6 | * | 22.6 | * |
|  | South Africa | 1 | 0 | 16.2 | * | * | 16.2 | * | 16.2 | * | 16.2 | * |
|  | Spain | 3 | 0 | 15.2 | 1.88 | 12.4 | 13.3 | 13.3 | 15.4 | 17 | 17 | 3.75 |
|  | Turkey | 2 | 0 | 15.6 | 2.01 | 12.9 | 14.2 | * | 15.6 | * | 17.1 | 2.84 |
|  | UK | 5 | 0 | 12.3 | 1.98 | 16.1 | 10.3 | 10.6 | 11.8 | 14.4 | 14.9 | 4.67 |
|  | USA | 1 | 1 | 12.8 | * | * | 12.8 | * | 12.8 | * | 12.8 | * |
|  | Uzbekistan | 1 | 0 | 11 | * | * | 11 | * | 11 | * | 11 | * |
|  | Vietnam | 1 | 0 | 33.8 | * | * | 33.8 | * | 33.8 | * | 33.8 | * |
| **Mn** | Argentina | 1 | 0 | 222 | * | * | 222 | * | 222 | * | 222 | * |
|  | Australia | 3 | 0 | 2780 | 3390 | 122 | 265 | 265 | 1450 | 6640 | 6640 | 6370 |
|  | Austria | 2 | 0 | 2870 | 3640 | 127 | 299 | * | 2870 | * | 5450 | 5150 |
|  | Belgium | 1 | 0 | 1590 | * | * | 1590 | * | 1590 | * | 1590 | * |
|  | Belize | 2 | 0 | 351 | 24.7 | 7.04 | 334 | * | 351 | * | 369 | 35 |
|  | Chile | 3 | 0 | 1290 | 650 | 50.5 | 810 | 810 | 1020 | 2030 | 2030 | 1220 |
|  | China | 2 | 0 | 101 | 9.42 | 9.33 | 94.4 | * | 101 | * | 108 | 13.3 |
|  | Croatia | 1 | 0 | 21800 | * | * | 21800 | * | 21800 | * | 21800 | * |
|  | Denmark | 15 | 0 | 360 | 187 | 51.8 | 85.8 | 226 | 338 | 458 | 814 | 728 |
|  | Finland | 1 | 0 | 1310 | * | * | 1310 | * | 1310 | * | 1310 | * |
|  | France | 2 | 0 | 5240 | 6430 | 123 | 689 | * | 5240 | * | 9780 | 9100 |
|  | Georgia | 1 | 0 | 5100 | * | * | 5100 | * | 5100 | * | 5100 | * |
|  | Germany | 20 | 0 | 2010 | 2520 | 125 | 126 | 357 | 1240 | 2190 | 9620 | 9500 |
|  | Greece | 9 | 0 | 1710 | 2360 | 138 | 272 | 348 | 714 | 2500 | 7450 | 7180 |
|  | India | 4 | 0 | 17700 | 16500 | 93.5 | 630 | 2100 | 18300 | 32600 | 33400 | 32800 |
|  | Italy | 7 | 0 | 282 | 289 | 103 | 78 | 101 | 195 | 369 | 899 | 821 |
|  | Japan | 1 | 0 | 787 | * | * | 787 | * | 787 | * | 787 | * |
|  | Madeira | 1 | 0 | 2390 | * | * | 2390 | * | 2390 | * | 2390 | * |
|  | Maledives | 1 | 0 | 746 | * | * | 746 | * | 746 | * | 746 | * |
|  | Mongolia | 1 | 0 | 270 | * | * | 270 | * | 270 | * | 270 | * |
|  | Morocco | 1 | 0 | 259 | * | * | 259 | * | 259 | * | 259 | * |
|  | Niue | 2 | 0 | 720 | 397 | 55.2 | 439 | * | 720 | * | 1000 | 562 |
|  | NZ | 245 | 0 | 5230 | 4920 | 94.2 | 190 | 1350 | 3450 | 8500 | 28900 | 28700 |
|  | Poland | 5 | 0 | 4000 | 4920 | 123 | 164 | 247 | 1340 | 9080 | 11300 | 11200 |
|  | Portugal | 1 | 0 | 595 | * | * | 595 | * | 595 | * | 595 | * |
|  | Russia | 4 | 0 | 2830 | 2790 | 98.7 | 477 | 705 | 2020 | 5750 | 6790 | 6320 |
|  | S. America | 1 | 0 | 826 | * | * | 826 | * | 826 | * | 826 | * |
|  | South Africa | 1 | 0 | 186 | * | * | 186 | * | 186 | * | 186 | * |
|  | Spain | 3 | 0 | 461 | 212 | 46.1 | 333 | 333 | 344 | 706 | 706 | 373 |
|  | Turkey | 2 | 0 | 450 | 101 | 22.5 | 378 | * | 450 | * | 521 | 143 |
|  | UK | 5 | 0 | 2230 | 2450 | 110 | 198 | 203 | 997 | 4880 | 5260 | 5070 |
|  | USA | 2 | 0 | 2050 | 2410 | 118 | 346 | * | 2050 | * | 3760 | 3410 |
|  | Uzbekistan | 1 | 0 | 329 | * | * | 329 | * | 329 | * | 329 | * |
|  | Vietnam | 1 | 0 | 702 | * | * | 702 | * | 702 | * | 702 | * |
| **Fe** | Argentina | 1 | 0 | 830 | * | * | 830 | * | 830 | * | 830 | * |
|  | Australia | 3 | 0 | 1390 | 1470 | 106 | 351 | 351 | 741 | 3070 | 3070 | 2720 |
|  | Austria | 2 | 0 | 1230 | 1030 | 84 | 498 | * | 1230 | * | 1950 | 1460 |
|  | Belgium | 0 | 1 | * | * | * | * | * | * | * | * | * |
|  | Belize | 2 | 0 | 799 | 281 | 35.2 | 600 | * | 799 | * | 998 | 398 |
|  | Chile | 3 | 0 | 1910 | 1620 | 84.7 | 56 | 56 | 2640 | 3040 | 3040 | 2980 |
|  | China | 2 | 0 | 573 | 22.3 | 3.89 | 557 | * | 573 | * | 589 | 31.5 |
|  | Croatia | 1 | 0 | 613 | * | * | 613 | * | 613 | * | 613 | * |
|  | Denmark | 15 | 0 | 1080 | 516 | 48 | 411 | 795 | 980 | 1380 | 2450 | 2040 |
|  | Finland | 1 | 0 | 1310 | * | * | 1310 | * | 1310 | * | 1310 | * |
|  | France | 1 | 1 | 719 | * | * | 719 | * | 719 | * | 719 | * |
|  | Georgia | 1 | 0 | 2290 | * | * | 2290 | * | 2290 | * | 2290 | * |
|  | Germany | 15 | 5 | 758 | 491 | 64.7 | 345 | 408 | 577 | 1050 | 1980 | 1630 |
|  | Greece | 9 | 0 | 5430 | 7530 | 139 | 897 | 2020 | 3110 | 5030 | 25200 | 24300 |
|  | India | 4 | 0 | 4420 | 4360 | 98.6 | 1480 | 1500 | 2740 | 9030 | 10700 | 9260 |
|  | Italy | 7 | 0 | 768 | 459 | 59.8 | 342 | 418 | 631 | 992 | 1690 | 1350 |
|  | Japan | 1 | 0 | 1020 | * | * | 1020 | * | 1020 | * | 1020 | * |
|  | Madeira | 1 | 0 | 1480 | * | * | 1480 | * | 1480 | * | 1480 | * |
|  | Maledives | 1 | 0 | 2180 | * | * | 2180 | * | 2180 | * | 2180 | * |
|  | Mongolia | 1 | 0 | 2430 | * | * | 2430 | * | 2430 | * | 2430 | * |
|  | Morocco | 1 | 0 | 1160 | * | * | 1160 | * | 1160 | * | 1160 | * |
|  | Niue | 2 | 0 | 1170 | 847 | 72.3 | 573 | * | 1170 | * | 1770 | 1200 |
|  | NZ | 226 | 19 | 1330 | 778 | 58.4 | 333 | 723 | 1120 | 1880 | 4280 | 3950 |
|  | Poland | 5 | 0 | 2790 | 2300 | 82.4 | 1350 | 1380 | 2080 | 4550 | 6830 | 5490 |
|  | Portugal | 1 | 0 | 1520 | * | * | 1520 | * | 1520 | * | 1520 | * |
|  | Russia | 4 | 0 | 1320 | 748 | 56.7 | 556 | 639 | 1240 | 2070 | 2230 | 1680 |
|  | S. America | 1 | 0 | 5620 | * | * | 5620 | * | 5620 | * | 5620 | * |
|  | South Africa | 1 | 0 | 2760 | * | * | 2760 | * | 2760 | * | 2760 | * |
|  | Spain | 3 | 0 | 2660 | 949 | 35.6 | 2030 | 2030 | 2210 | 3760 | 3760 | 1730 |
|  | Turkey | 2 | 0 | 1960 | 47.8 | 2.44 | 1930 | * | 1960 | * | 2000 | 67.7 |
|  | UK | 5 | 0 | 619 | 365 | 59 | 364 | 370 | 461 | 946 | 1240 | 873 |
|  | USA | 2 | 0 | 536 | 132 | 24.5 | 443 | * | 536 | * | 630 | 186 |
|  | Uzbekistan | 1 | 0 | 458 | * | * | 458 | * | 458 | * | 458 | * |
|  | Vietnam | 1 | 0 | 2070 | * | * | 2070 | * | 2070 | * | 2070 | * |
| **Co** | Argentina | 0 | 1 | * | * | * | * | * | * | * | * | * |
|  | Australia | 1 | 2 | 8.36 | * | * | 8.36 | * | 8.36 | * | 8.36 | * |
|  | Austria | 1 | 1 | 34.3 | * | * | 34.3 | * | 34.3 | * | 34.3 | * |
|  | Belgium | 1 | 0 | 15.8 | * | * | 15.8 | * | 15.8 | * | 15.8 | * |
|  | Belize | 1 | 1 | 8.88 | * | * | 8.88 | * | 8.88 | * | 8.88 | * |
|  | Chile | 1 | 2 | 29.9 | * | * | 29.9 | * | 29.9 | * | 29.9 | * |
|  | China | 0 | 2 | * | * | * | * | * | * | * | * | * |
|  | Croatia | 0 | 1 | * | * | * | * | * | * | * | * | * |
|  | Denmark | 1 | 14 | 7.62 | * | * | 7.62 | * | 7.62 | * | 7.62 | * |
|  | Finland | 1 | 0 | 8.09 | * | * | 8.09 | * | 8.09 | * | 8.09 | * |
|  | France | 0 | 2 | * | * | * | * | * | * | * | * | * |
|  | Georgia | 0 | 1 | * | * | * | * | * | * | * | * | * |
|  | Germany | 3 | 17 | 15.1 | 12.8 | 84.7 | 6.68 | 6.68 | 8.8 | 29.8 | 29.8 | 23.1 |
|  | Greece | 3 | 6 | 14.7 | 12.8 | 87.2 | 7.26 | 7.26 | 7.34 | 29.5 | 29.5 | 22.3 |
|  | India | 2 | 2 | 14.1 | 9.1 | 64.4 | 7.7 | * | 14.1 | * | 20.6 | 12.9 |
|  | Italy | 1 | 6 | 7.85 | * | * | 7.85 | * | 7.85 | * | 7.85 | * |
|  | Japan | 0 | 1 | * | * | * | * | * | * | * | * | * |
|  | Madeira | 1 | 0 | 132 | * | * | 132 | * | 132 | * | 132 | * |
|  | Maledives | 1 | 0 | 7.2 | * | * | 7.2 | * | 7.2 | * | 7.2 | * |
|  | Mongolia | 0 | 1 | * | * | * | * | * | * | * | * | * |
|  | Morocco | 0 | 1 | * | * | * | * | * | * | * | * | * |
|  | Niue | 0 | 2 | * | * | * | * | * | * | * | * | * |
|  | NZ | 77 | 168 | 7.63 | 5.15 | 67.6 | 1.24 | 3.95 | 6.83 | 9.81 | 24.5 | 23.3 |
|  | Poland | 1 | 4 | 24.9 | * | * | 24.9 | * | 24.9 | * | 24.9 | * |
|  | Portugal | 0 | 1 | * | * | * | * | * | * | * | * | * |
|  | Russia | 1 | 3 | 16.4 | * | * | 16.4 | * | 16.4 | * | 16.4 | * |
|  | S. America | 1 | 0 | 7.55 | * | * | 7.55 | * | 7.55 | * | 7.55 | * |
|  | South Africa | 0 | 1 | * | * | * | * | * | * | * | * | * |
|  | Spain | 2 | 1 | 12.9 | 0.111 | 0.86 | 12.8 | * | 12.9 | * | 13 | 0.157 |
|  | Turkey | 0 | 2 | * | * | * | * | * | * | * | * | * |
|  | UK | 2 | 3 | 9.75 | 2.75 | 28.2 | 7.81 | * | 9.75 | * | 11.7 | 3.89 |
|  | USA | 1 | 1 | 8.83 | * | * | 8.83 | * | 8.83 | * | 8.83 | * |
|  | Uzbekistan | 0 | 1 | * | * | * | * | * | * | * | * | * |
|  | Vietnam | 0 | 1 | * | * | * | * | * | * | * | * | * |
| **Ni** | Argentina | 1 | 0 | 14.2 | * | * | 14.2 | * | 14.2 | * | 14.2 | * |
|  | Australia | 3 | 0 | 34.2 | 24.6 | 72 | 19.5 | 19.5 | 20.5 | 62.6 | 62.6 | 43.1 |
|  | Austria | 2 | 0 | 394 | 509 | 129 | 34 | * | 394 | * | 754 | 720 |
|  | Belgium | 1 | 0 | 14.7 | * | * | 14.7 | * | 14.7 | * | 14.7 | * |
|  | Belize | 1 | 1 | 14 | * | * | 14 | * | 14 | * | 14 | * |
|  | Chile | 3 | 0 | 41.7 | 46.9 | 113 | -5.7 | -5.7 | 42.7 | 88.2 | 88.2 | 93.9 |
|  | China | 1 | 1 | 23.6 | * | * | 23.6 | * | 23.6 | * | 23.6 | * |
|  | Croatia | 1 | 0 | 95.1 | * | * | 95.1 | * | 95.1 | * | 95.1 | * |
|  | Denmark | 14 | 1 | 8.54 | 5.2 | 60.9 | 2.57 | 5.31 | 7.42 | 9.64 | 23.6 | 21 |
|  | Finland | 1 | 0 | 41.5 | * | * | 41.5 | * | 41.5 | * | 41.5 | * |
|  | France | 1 | 1 | 36.8 | * | * | 36.8 | * | 36.8 | * | 36.8 | * |
|  | Georgia | 1 | 0 | 20.6 | * | * | 20.6 | * | 20.6 | * | 20.6 | * |
|  | Germany | 17 | 3 | 37.5 | 28.1 | 75 | 13.4 | 19.4 | 29.2 | 46.3 | 129 | 116 |
|  | Greece | 9 | 0 | 188 | 302 | 161 | 35 | 73 | 88 | 126 | 991 | 956 |
|  | India | 4 | 0 | 100 | 84.6 | 84.3 | 24.7 | 31.4 | 80.7 | 189 | 216 | 191 |
|  | Italy | 7 | 0 | 72.5 | 61.4 | 84.7 | 13.4 | 29.7 | 51.1 | 122 | 188 | 174 |
|  | Japan | 1 | 0 | 47.7 | * | * | 47.7 | * | 47.7 | * | 47.7 | * |
|  | Madeira | 1 | 0 | 178 | * | * | 178 | * | 178 | * | 178 | * |
|  | Maledives | 1 | 0 | 45.1 | * | * | 45.1 | * | 45.1 | * | 45.1 | * |
|  | Mongolia | 1 | 0 | 25.6 | * | * | 25.6 | * | 25.6 | * | 25.6 | * |
|  | Morocco | 0 | 1 | * | * | * | * | * | * | * | * | * |
|  | Niue | 2 | 0 | 2.6 | 10.9 | 419 | -5.1 | * | 2.6 | * | 10.3 | 15.4 |
|  | NZ | 238 | 7 | 35.4 | 102 | 288 | 0.15 | 10.3 | 17 | 32.5 | 1200 | 1200 |
|  | Poland | 5 | 0 | 152 | 269 | 177 | 14 | 21 | 31 | 343 | 631 | 617 |
|  | Portugal | 0 | 1 | * | * | * | * | * | * | * | * | * |
|  | Russia | 3 | 1 | 20.8 | 5.53 | 26.6 | 15.7 | 15.7 | 20.1 | 26.7 | 26.7 | 11 |
|  | S. America | 1 | 0 | 19.8 | * | * | 19.8 | * | 19.8 | * | 19.8 | * |
|  | South Africa | 1 | 0 | 15.1 | * | * | 15.1 | * | 15.1 | * | 15.1 | * |
|  | Spain | 3 | 0 | 64 | 47.2 | 73.8 | 15.7 | 15.7 | 66.3 | 110 | 110 | 94.4 |
|  | Turkey | 2 | 0 | 28.7 | 8.86 | 30.8 | 22.5 | * | 28.7 | * | 35 | 12.5 |
|  | UK | 5 | 0 | 24.7 | 5.53 | 22.4 | 17.5 | 20.2 | 23.6 | 29.8 | 32.4 | 14.9 |
|  | USA | 1 | 1 | 34.8 | * | * | 34.8 | * | 34.8 | * | 34.8 | * |
|  | Uzbekistan | 1 | 0 | 14.1 | * | * | 14.1 | * | 14.1 | * | 14.1 | * |
|  | Vietnam | 1 | 0 | 13.6 | * | * | 13.6 | * | 13.6 | * | 13.6 | * |
| **Cu** | Argentina | 1 | 0 | 115 | * | * | 115 | * | 115 | * | 115 | * |
|  | Australia | 3 | 0 | 117 | 38.6 | 33 | 83.8 | 83.8 | 108 | 159 | 159 | 75.6 |
|  | Austria | 2 | 0 | 587 | 458 | 78.1 | 263 | * | 587 | * | 911 | 648 |
|  | Belgium | 1 | 0 | 215 | * | * | 215 | * | 215 | * | 215 | * |
|  | Belize | 2 | 0 | 39.4 | 16 | 40.7 | 28 | * | 39.4 | * | 50.7 | 22.6 |
|  | Chile | 3 | 0 | 368 | 268 | 73 | 159 | 159 | 273 | 670 | 670 | 511 |
|  | China | 2 | 0 | 2.6 | 44.3 | 1700 | -28.7 | * | 2.6 | * | 33.9 | 62.6 |
|  | Croatia | 1 | 0 | 298 | * | * | 298 | * | 298 | * | 298 | * |
|  | Denmark | 15 | 0 | 328 | 80.5 | 24.5 | 138 | 285 | 342 | 388 | 421 | 284 |
|  | Finland | 1 | 0 | 68.7 | * | * | 68.7 | * | 68.7 | * | 68.7 | * |
|  | France | 2 | 0 | 97.2 | 79.2 | 81.5 | 41.2 | * | 97.2 | * | 153 | 112 |
|  | Georgia | 1 | 0 | 50 | * | * | 50 | * | 50 | * | 50 | * |
|  | Germany | 20 | 0 | 190 | 158 | 83.1 | 7 | 49 | 138 | 296 | 576 | 569 |
|  | Greece | 9 | 0 | 541 | 516 | 95.3 | 192 | 217 | 329 | 676 | 1810 | 1620 |
|  | India | 4 | 0 | 346 | 295 | 85.1 | 30 | 63 | 369 | 607 | 617 | 587 |
|  | Italy | 7 | 0 | 273 | 272 | 99.8 | 37 | 66 | 152 | 598 | 720 | 684 |
|  | Japan | 1 | 0 | 78.6 | * | * | 78.6 | * | 78.6 | * | 78.6 | * |
|  | Madeira | 1 | 0 | 125 | * | * | 125 | * | 125 | * | 125 | * |
|  | Maledives | 1 | 0 | 102 | * | * | 102 | * | 102 | * | 102 | * |
|  | Mongolia | 1 | 0 | 53.9 | * | * | 53.9 | * | 53.9 | * | 53.9 | * |
|  | Morocco | 1 | 0 | 63.4 | * | * | 63.4 | * | 63.4 | * | 63.4 | * |
|  | Niue | 2 | 0 | 570 | 138 | 24.1 | 473 | * | 570 | * | 667 | 194 |
|  | NZ | 245 | 0 | 471 | 334 | 70.8 | 65.6 | 225 | 393 | 628 | 2140 | 2070 |
|  | Poland | 5 | 0 | 553 | 642 | 116 | 22 | 69 | 331 | 1150 | 1610 | 1580 |
|  | Portugal | 1 | 0 | 264 | * | * | 264 | * | 264 | * | 264 | * |
|  | Russia | 4 | 0 | 192 | 164 | 85.4 | 34.2 | 61 | 156 | 359 | 422 | 388 |
|  | S. America | 1 | 0 | 164 | * | * | 164 | * | 164 | * | 164 | * |
|  | South Africa | 1 | 0 | 10.1 | * | * | 10.1 | * | 10.1 | * | 10.1 | * |
|  | Spain | 3 | 0 | 522 | 465 | 89 | 166 | 166 | 352 | 1050 | 1050 | 882 |
|  | Turkey | 2 | 0 | 141 | 36.6 | 26 | 115 | * | 141 | * | 167 | 51.7 |
|  | UK | 5 | 0 | 158 | 87.1 | 55.1 | 76.8 | 96.5 | 144 | 227 | 305 | 229 |
|  | USA | 2 | 0 | 156 | 174 | 112 | 33 | * | 156 | * | 279 | 246 |
|  | Uzbekistan | 1 | 0 | 154 | * | * | 154 | * | 154 | * | 154 | * |
|  | Vietnam | 1 | 0 | 55.9 | * | * | 55.9 | * | 55.9 | * | 55.9 | * |
| **Zn** | Argentina | 1 | 0 | 709 | * | * | 709 | * | 709 | * | 709 | * |
|  | Australia | 3 | 0 | 57600 | 98700 | 171 | 433 | 433 | 847 | 172000 | 172000 | 171000 |
|  | Austria | 2 | 0 | 1430 | 1240 | 86.7 | 552 | * | 1430 | * | 2300 | 1750 |
|  | Belgium | 1 | 0 | 717 | * | * | 717 | * | 717 | * | 717 | * |
|  | Belize | 2 | 0 | 549 | 109 | 19.8 | 472 | * | 549 | * | 625 | 153 |
|  | Chile | 3 | 0 | 941 | 73.1 | 7.78 | 895 | 895 | 902 | 1020 | 1020 | 130 |
|  | China | 2 | 0 | 261 | 192 | 73.6 | 125 | * | 261 | * | 397 | 272 |
|  | Croatia | 1 | 0 | 1190 | * | * | 1190 | * | 1190 | * | 1190 | * |
|  | Denmark | 15 | 0 | 571 | 182 | 31.8 | 320 | 453 | 557 | 671 | 1070 | 754 |
|  | Finland | 1 | 0 | 481 | * | * | 481 | * | 481 | * | 481 | * |
|  | France | 2 | 0 | 1080 | 1020 | 94.2 | 361 | * | 1080 | * | 1800 | 1440 |
|  | Georgia | 1 | 0 | 412 | * | * | 412 | * | 412 | * | 412 | * |
|  | Germany | 20 | 0 | 747 | 533 | 71.3 | 228 | 430 | 566 | 897 | 2430 | 2200 |
|  | Greece | 9 | 0 | 1550 | 663 | 42.7 | 807 | 1100 | 1420 | 1860 | 3040 | 2240 |
|  | India | 4 | 0 | 1910 | 1410 | 73.9 | 835 | 853 | 1480 | 3410 | 3860 | 3030 |
|  | Italy | 7 | 0 | 973 | 528 | 54.3 | 375 | 573 | 741 | 1460 | 1750 | 1380 |
|  | Japan | 1 | 0 | 615 | * | * | 615 | * | 615 | * | 615 | * |
|  | Madeira | 1 | 0 | 1090 | * | * | 1090 | * | 1090 | * | 1090 | * |
|  | Maledives | 1 | 0 | 1130 | * | * | 1130 | * | 1130 | * | 1130 | * |
|  | Mongolia | 1 | 0 | 10400 | * | * | 10400 | * | 10400 | * | 10400 | * |
|  | Morocco | 1 | 0 | 675 | * | * | 675 | * | 675 | * | 675 | * |
|  | Niue | 2 | 0 | 251 | 39.6 | 15.8 | 223 | * | 251 | * | 279 | 56 |
|  | NZ | 245 | 0 | 1020 | 670 | 66 | 230 | 621 | 904 | 1160 | 5970 | 5740 |
|  | Poland | 5 | 0 | 1720 | 1730 | 101 | 449 | 553 | 882 | 3290 | 4630 | 4180 |
|  | Portugal | 1 | 0 | 1480 | * | * | 1480 | * | 1480 | * | 1480 | * |
|  | Russia | 4 | 0 | 1640 | 1330 | 80.9 | 335 | 574 | 1370 | 2980 | 3490 | 3150 |
|  | S. America | 1 | 0 | 1550 | * | * | 1550 | * | 1550 | * | 1550 | * |
|  | South Africa | 1 | 0 | 723 | * | * | 723 | * | 723 | * | 723 | * |
|  | Spain | 3 | 0 | 2210 | 1750 | 79.2 | 916 | 916 | 1520 | 4210 | 4210 | 3290 |
|  | Turkey | 2 | 0 | 2300 | 2190 | 95.3 | 749 | * | 2300 | * | 3850 | 3100 |
|  | UK | 5 | 0 | 2080 | 1640 | 78.8 | 916 | 939 | 1150 | 3680 | 4710 | 3800 |
|  | USA | 2 | 0 | 783 | 426 | 54.4 | 482 | * | 783 | * | 1080 | 602 |
|  | Uzbekistan | 1 | 0 | 490 | * | * | 490 | * | 490 | * | 490 | * |
|  | Vietnam | 1 | 0 | 821 | * | * | 821 | * | 821 | * | 821 | * |
| **Rb** | Argentina | 1 | 0 | 181 | * | * | 181 | * | 181 | * | 181 | * |
|  | Australia | 3 | 0 | 2810 | 1740 | 61.9 | 1570 | 1570 | 2060 | 4790 | 4790 | 3220 |
|  | Austria | 2 | 0 | 5730 | 7120 | 124 | 699 | * | 5730 | * | 10800 | 10100 |
|  | Belgium | 1 | 0 | 388 | * | * | 388 | * | 388 | * | 388 | * |
|  | Belize | 2 | 0 | 450 | 476 | 106 | 114 | * | 450 | * | 787 | 673 |
|  | Chile | 3 | 0 | 2560 | 512 | 20 | 2190 | 2190 | 2350 | 3140 | 3140 | 956 |
|  | China | 2 | 0 | 150 | 78.3 | 52.2 | 94.7 | * | 150 | * | 206 | 111 |
|  | Croatia | 1 | 0 | 6510 | * | * | 6510 | * | 6510 | * | 6510 | * |
|  | Denmark | 15 | 0 | 554 | 267 | 48.1 | 233 | 402 | 476 | 612 | 1310 | 1080 |
|  | Finland | 1 | 0 | 1020 | * | * | 1020 | * | 1020 | * | 1020 | * |
|  | France | 2 | 0 | 2610 | 3450 | 132 | 171 | * | 2610 | * | 5050 | 4880 |
|  | Georgia | 1 | 0 | 3620 | * | * | 3620 | * | 3620 | * | 3620 | * |
|  | Germany | 20 | 0 | 2000 | 4430 | 222 | 134 | 277 | 945 | 1800 | 20600 | 20400 |
|  | Greece | 9 | 0 | 2650 | 1990 | 75.2 | 552 | 876 | 2410 | 4210 | 6250 | 5700 |
|  | India | 4 | 0 | 8660 | 6320 | 73 | 676 | 2180 | 9550 | 14200 | 14800 | 14200 |
|  | Italy | 7 | 0 | 1150 | 1250 | 109 | 157 | 234 | 500 | 1920 | 3560 | 3400 |
|  | Japan | 1 | 0 | 3350 | * | * | 3350 | * | 3350 | * | 3350 | * |
|  | Madeira | 1 | 0 | 3300 | * | * | 3300 | * | 3300 | * | 3300 | * |
|  | Maledives | 1 | 0 | 899 | * | * | 899 | * | 899 | * | 899 | * |
|  | Mongolia | 1 | 0 | 149 | * | * | 149 | * | 149 | * | 149 | * |
|  | Morocco | 1 | 0 | 111 | * | * | 111 | * | 111 | * | 111 | * |
|  | Niue | 2 | 0 | 444 | 8 | 1.8 | 438 | * | 444 | * | 449 | 11.3 |
|  | NZ | 245 | 0 | 3790 | 1920 | 50.6 | 179 | 2360 | 3470 | 4960 | 13400 | 13200 |
|  | Poland | 5 | 0 | 3460 | 3080 | 89.2 | 197 | 449 | 3230 | 6590 | 6900 | 6700 |
|  | Portugal | 1 | 0 | 3880 | * | * | 3880 | * | 3880 | * | 3880 | * |
|  | Russia | 4 | 0 | 590 | 478 | 81 | 172 | 177 | 551 | 1040 | 1090 | 913 |
|  | S. America | 1 | 0 | 709 | * | * | 709 | * | 709 | * | 709 | * |
|  | South Africa | 1 | 0 | 222 | * | * | 222 | * | 222 | * | 222 | * |
|  | Spain | 3 | 0 | 2140 | 2200 | 103 | 455 | 455 | 1350 | 4630 | 4630 | 4180 |
|  | Turkey | 2 | 0 | 361 | 49.9 | 13.8 | 326 | * | 361 | * | 396 | 70.6 |
|  | UK | 5 | 0 | 867 | 772 | 89 | 203 | 222 | 495 | 1700 | 1820 | 1610 |
|  | USA | 2 | 0 | 686 | 805 | 117 | 117 | * | 686 | * | 1260 | 1140 |
|  | Uzbekistan | 1 | 0 | 3150 | * | * | 3150 | * | 3150 | * | 3150 | * |
|  | Vietnam | 1 | 0 | 439 | * | * | 439 | * | 439 | * | 439 | * |
| **Sr** | Argentina | 1 | 0 | 111 | * | * | 111 | * | 111 | * | 111 | * |
|  | Australia | 3 | 0 | 518 | 259 | 49.9 | 274 | 274 | 492 | 789 | 789 | 515 |
|  | Austria | 2 | 0 | 93.6 | 45.4 | 48.5 | 61.5 | * | 93.6 | * | 126 | 64.2 |
|  | Belgium | 1 | 0 | 69.6 | * | * | 69.6 | * | 69.6 | * | 69.6 | * |
|  | Belize | 2 | 0 | 133 | 133 | 100 | 38.6 | * | 133 | * | 227 | 188 |
|  | Chile | 3 | 0 | 9550 | 13800 | 145 | 84 | 84 | 3130 | 25400 | 25400 | 25400 |
|  | China | 2 | 0 | 144 | 89.6 | 62.2 | 80.6 | * | 144 | * | 207 | 127 |
|  | Croatia | 1 | 0 | 391 | * | * | 391 | * | 391 | * | 391 | * |
|  | Denmark | 15 | 0 | 152 | 47.8 | 31.5 | 77.8 | 117 | 142 | 183 | 246 | 168 |
|  | Finland | 1 | 0 | 168 | * | * | 168 | * | 168 | * | 168 | * |
|  | France | 2 | 0 | 205 | 249 | 121 | 29 | * | 205 | * | 381 | 352 |
|  | Georgia | 1 | 0 | 333 | * | * | 333 | * | 333 | * | 333 | * |
|  | Germany | 20 | 0 | 125 | 80.1 | 64 | 31.7 | 68.9 | 104 | 158 | 357 | 325 |
|  | Greece | 9 | 0 | 187 | 83.4 | 44.6 | 89.3 | 122 | 143 | 253 | 340 | 251 |
|  | India | 4 | 0 | 537 | 345 | 64.3 | 107 | 206 | 546 | 858 | 948 | 841 |
|  | Italy | 7 | 0 | 274 | 236 | 86.1 | 33.2 | 53.9 | 229 | 469 | 673 | 640 |
|  | Japan | 1 | 0 | 256 | * | * | 256 | * | 256 | * | 256 | * |
|  | Madeira | 1 | 0 | 372 | * | * | 372 | * | 372 | * | 372 | * |
|  | Maledives | 1 | 0 | 252 | * | * | 252 | * | 252 | * | 252 | * |
|  | Mongolia | 1 | 0 | 132 | * | * | 132 | * | 132 | * | 132 | * |
|  | Morocco | 1 | 0 | 128 | * | * | 128 | * | 128 | * | 128 | * |
|  | Niue | 2 | 0 | 145 | 17.9 | 12.4 | 132 | * | 145 | * | 158 | 25.3 |
|  | NZ | 245 | 0 | 194 | 117 | 60.5 | 43 | 114 | 156 | 247 | 878 | 835 |
|  | Poland | 5 | 0 | 158 | 58.2 | 36.8 | 97.7 | 108 | 146 | 215 | 243 | 146 |
|  | Portugal | 1 | 0 | 55.4 | * | * | 55.4 | * | 55.4 | * | 55.4 | * |
|  | Russia | 4 | 0 | 146 | 121 | 83.2 | 25.7 | 44 | 123 | 270 | 311 | 286 |
|  | S. America | 1 | 0 | 833 | * | * | 833 | * | 833 | * | 833 | * |
|  | South Africa | 1 | 0 | 92.6 | * | * | 92.6 | * | 92.6 | * | 92.6 | * |
|  | Spain | 3 | 0 | 444 | 288 | 64.8 | 115 | 115 | 572 | 645 | 645 | 531 |
|  | Turkey | 2 | 0 | 247 | 182 | 73.7 | 118 | * | 247 | * | 375 | 257 |
|  | UK | 5 | 0 | 83.9 | 16.7 | 19.9 | 59.7 | 67.9 | 86.2 | 98.8 | 102 | 41.9 |
|  | USA | 2 | 0 | 238 | 10.8 | 4.51 | 231 | * | 238 | * | 246 | 15.2 |
|  | Uzbekistan | 1 | 0 | 58.2 | * | * | 58.2 | * | 58.2 | * | 58.2 | * |
|  | Vietnam | 1 | 0 | 407 | * | * | 407 | * | 407 | * | 407 | * |
| **Cd** | Argentina | 0 | 1 | * | * | * | * | * | * | * | * | * |
|  | Australia | 1 | 2 | 8.41 | * | * | 8.41 | * | 8.41 | * | 8.41 | * |
|  | Austria | 1 | 1 | 6.85 | * | * | 6.85 | * | 6.85 | * | 6.85 | * |
|  | Belgium | 0 | 1 | * | * | * | * | * | * | * | * | * |
|  | Belize | 0 | 2 | * | * | * | * | * | * | * | * | * |
|  | Chile | 0 | 3 | * | * | * | * | * | * | * | * | * |
|  | China | 0 | 2 | * | * | * | * | * | * | * | * | * |
|  | Croatia | 0 | 1 | * | * | * | * | * | * | * | * | * |
|  | Denmark | 0 | 15 | * | * | * | * | * | * | * | * | * |
|  | Finland | 0 | 1 | * | * | * | * | * | * | * | * | * |
|  | France | 0 | 2 | * | * | * | * | * | * | * | * | * |
|  | Georgia | 0 | 1 | * | * | * | * | * | * | * | * | * |
|  | Germany | 0 | 20 | * | * | * | * | * | * | * | * | * |
|  | Greece | 2 | 7 | 11.8 | 7.34 | 62.2 | 6.62 | * | 11.8 | * | 17 | 10.4 |
|  | India | 0 | 4 | * | * | * | * | * | * | * | * | * |
|  | Italy | 0 | 7 | * | * | * | * | * | * | * | * | * |
|  | Japan | 0 | 1 | * | * | * | * | * | * | * | * | * |
|  | Madeira | 0 | 1 | * | * | * | * | * | * | * | * | * |
|  | Maledives | 0 | 1 | * | * | * | * | * | * | * | * | * |
|  | Mongolia | 0 | 1 | * | * | * | * | * | * | * | * | * |
|  | Morocco | 0 | 1 | * | * | * | * | * | * | * | * | * |
|  | Niue | 0 | 2 | * | * | * | * | * | * | * | * | * |
|  | NZ | 43 | 202 | 11.3 | 13.5 | 119 | 1.45 | 2.06 | 6.06 | 17.4 | 51.6 | 50.2 |
|  | Poland | 2 | 3 | 39.1 | 13.4 | 34.4 | 29.6 | * | 39.1 | * | 48.6 | 19 |
|  | Portugal | 1 | 0 | 6.42 | * | * | 6.42 | * | 6.42 | * | 6.42 | * |
|  | Russia | 0 | 4 | * | * | * | * | * | * | * | * | * |
|  | S. America | 0 | 1 | * | * | * | * | * | * | * | * | * |
|  | South Africa | 0 | 1 | * | * | * | * | * | * | * | * | * |
|  | Spain | 0 | 3 | * | * | * | * | * | * | * | * | * |
|  | Turkey | 0 | 2 | * | * | * | * | * | * | * | * | * |
|  | UK | 0 | 5 | * | * | * | * | * | * | * | * | * |
|  | USA | 0 | 2 | * | * | * | * | * | * | * | * | * |
|  | Uzbekistan | 0 | 1 | * | * | * | * | * | * | * | * | * |
|  | Vietnam | 0 | 1 | * | * | * | * | * | * | * | * | * |
| **Cs** | Argentina | 0 | 1 | * | * | * | * | * | * | * | * | * |
|  | Australia | 0 | 3 | * | * | * | * | * | * | * | * | * |
|  | Austria | 1 | 1 | 112 | * | * | 112 | * | 112 | * | 112 | * |
|  | Belgium | 0 | 1 | * | * | * | * | * | * | * | * | * |
|  | Belize | 1 | 1 | 11.4 | * | * | 11.4 | * | 11.4 | * | 11.4 | * |
|  | Chile | 1 | 2 | 9.52 | * | * | 9.52 | * | 9.52 | * | 9.52 | * |
|  | China | 0 | 2 | * | * | * | * | * | * | * | * | * |
|  | Croatia | 1 | 0 | 16.1 | * | * | 16.1 | * | 16.1 | * | 16.1 | * |
|  | Denmark | 0 | 15 | * | * | * | * | * | * | * | * | * |
|  | Finland | 0 | 1 | * | * | * | * | * | * | * | * | * |
|  | France | 0 | 2 | * | * | * | * | * | * | * | * | * |
|  | Georgia | 0 | 1 | * | * | * | * | * | * | * | * | * |
|  | Germany | 1 | 19 | 63.9 | * | * | 63.9 | * | 63.9 | * | 63.9 | * |
|  | Greece | 5 | 4 | 18.9 | 11.7 | 61.7 | 9.41 | 10.7 | 12.4 | 30.4 | 37.5 | 28.1 |
|  | India | 3 | 1 | 68.1 | 55.1 | 80.9 | 21.5 | 21.5 | 54 | 129 | 129 | 107 |
|  | Italy | 0 | 7 | * | * | * | * | * | * | * | * | * |
|  | Japan | 0 | 1 | * | * | * | * | * | * | * | * | * |
|  | Madeira | 0 | 1 | * | * | * | * | * | * | * | * | * |
|  | Maledives | 0 | 1 | * | * | * | * | * | * | * | * | * |
|  | Mongolia | 0 | 1 | * | * | * | * | * | * | * | * | * |
|  | Morocco | 0 | 1 | * | * | * | * | * | * | * | * | * |
|  | Niue | 0 | 2 | * | * | * | * | * | * | * | * | * |
|  | NZ | 197 | 48 | 32.7 | 47.1 | 144 | 0.67 | 13.8 | 21.7 | 36.9 | 496 | 495 |
|  | Poland | 2 | 3 | 149 | 147 | 98.6 | 45 | * | 149 | * | 253 | 208 |
|  | Portugal | 0 | 1 | * | * | * | * | * | * | * | * | * |
|  | Russia | 0 | 4 | * | * | * | * | * | * | * | * | * |
|  | S. America | 0 | 1 | * | * | * | * | * | * | * | * | * |
|  | South Africa | 0 | 1 | * | * | * | * | * | * | * | * | * |
|  | Spain | 0 | 3 | * | * | * | * | * | * | * | * | * |
|  | Turkey | 0 | 2 | * | * | * | * | * | * | * | * | * |
|  | UK | 2 | 3 | 32.5 | 1.76 | 5.44 | 31.2 | * | 32.5 | * | 33.7 | 2.49 |
|  | USA | 0 | 2 | * | * | * | * | * | * | * | * | * |
|  | Uzbekistan | 1 | 0 | 21.1 | * | * | 21.1 | * | 21.1 | * | 21.1 | * |
|  | Vietnam | 0 | 1 | * | * | * | * | * | * | * | * | * |
| **Ba** | Argentina | 1 | 0 | 15 | * | * | 15 | * | 15 | * | 15 | * |
|  | Australia | 3 | 0 | 311 | 224 | 71.9 | 171 | 171 | 194 | 570 | 570 | 399 |
|  | Austria | 2 | 0 | 60.3 | 19.8 | 32.8 | 46.4 | * | 60.3 | * | 74.3 | 28 |
|  | Belgium | 1 | 0 | 43.2 | * | * | 43.2 | * | 43.2 | * | 43.2 | * |
|  | Belize | 2 | 0 | 67.1 | 23.5 | 35 | 50.5 | * | 67.1 | * | 83.8 | 33.3 |
|  | Chile | 3 | 0 | 5400 | 7840 | 145 | 68 | 68 | 1720 | 14400 | 14400 | 14300 |
|  | China | 2 | 0 | 32.9 | 16.1 | 49.1 | 21.5 | * | 32.9 | * | 44.3 | 22.8 |
|  | Croatia | 1 | 0 | 1130 | * | * | 1130 | * | 1130 | * | 1130 | * |
|  | Denmark | 15 | 0 | 71.8 | 29.3 | 40.9 | 39.6 | 55.7 | 65.2 | 76.1 | 158 | 118 |
|  | Finland | 1 | 0 | 82 | * | * | 82 | * | 82 | * | 82 | * |
|  | France | 2 | 0 | 111 | 143 | 129 | 10 | * | 111 | * | 213 | 203 |
|  | Georgia | 1 | 0 | 399 | * | * | 399 | * | 399 | * | 399 | * |
|  | Germany | 20 | 0 | 79 | 88.6 | 112 | 11.8 | 19.1 | 71.3 | 83.1 | 402 | 391 |
|  | Greece | 9 | 0 | 87.4 | 71.4 | 81.7 | 27.1 | 31.4 | 77.4 | 107 | 259 | 232 |
|  | India | 4 | 0 | 523 | 453 | 86.5 | 115 | 124 | 509 | 937 | 961 | 846 |
|  | Italy | 7 | 0 | 51.6 | 24.1 | 46.7 | 12.5 | 40.3 | 50.4 | 80.5 | 81.8 | 69.3 |
|  | Japan | 1 | 0 | 256 | * | * | 256 | * | 256 | * | 256 | * |
|  | Madeira | 1 | 0 | 338 | * | * | 338 | * | 338 | * | 338 | * |
|  | Maledives | 1 | 0 | 176 | * | * | 176 | * | 176 | * | 176 | * |
|  | Mongolia | 1 | 0 | 37 | * | * | 37 | * | 37 | * | 37 | * |
|  | Morocco | 1 | 0 | 38.9 | * | * | 38.9 | * | 38.9 | * | 38.9 | * |
|  | Niue | 0 | 2 | * | * | * | * | * | * | * | * | * |
|  | NZ | 245 | 0 | 181 | 119 | 65.5 | 47.5 | 109 | 160 | 214 | 1100 | 1060 |
|  | Poland | 5 | 0 | 118 | 71.6 | 60.9 | 30.6 | 45.8 | 141 | 178 | 209 | 178 |
|  | Portugal | 1 | 0 | 42.2 | * | * | 42.2 | * | 42.2 | * | 42.2 | * |
|  | Russia | 4 | 0 | 105 | 154 | 146 | 11.9 | 13 | 37.4 | 265 | 334 | 322 |
|  | S. America | 1 | 0 | 436 | * | * | 436 | * | 436 | * | 436 | * |
|  | South Africa | 1 | 0 | 35.3 | * | * | 35.3 | * | 35.3 | * | 35.3 | * |
|  | Spain | 3 | 0 | 62 | 64.4 | 104 | 21.8 | 21.8 | 27.8 | 136 | 136 | 115 |
|  | Turkey | 2 | 0 | 48.6 | 1.76 | 3.63 | 47.3 | * | 48.6 | * | 49.8 | 2.5 |
|  | UK | 5 | 0 | 67.5 | 47.5 | 70.4 | 13.3 | 16 | 92.8 | 106 | 112 | 98.5 |
|  | USA | 2 | 0 | 163 | 109 | 66.9 | 86 | * | 163 | * | 240 | 154 |
|  | Uzbekistan | 1 | 0 | 208 | * | * | 208 | * | 208 | * | 208 | * |
|  | Vietnam | 1 | 0 | 104 | * | * | 104 | * | 104 | * | 104 | * |
| **Tl** | Argentina | 0 | 1 | * | * | * | * | * | * | * | * | * |
|  | Australia | 0 | 3 | * | * | * | * | * | * | * | * | * |
|  | Austria | 0 | 2 | * | * | * | * | * | * | * | * | * |
|  | Belgium | 0 | 1 | * | * | * | * | * | * | * | * | * |
|  | Belize | 0 | 2 | * | * | * | * | * | * | * | * | * |
|  | Chile | 1 | 2 | 10 | * | * | 10 | * | 10 | * | 10 | * |
|  | China | 0 | 2 | * | * | * | * | * | * | * | * | * |
|  | Croatia | 0 | 1 | * | * | * | * | * | * | * | * | * |
|  | Denmark | 0 | 15 | * | * | * | * | * | * | * | * | * |
|  | Finland | 0 | 1 | * | * | * | * | * | * | * | * | * |
|  | France | 0 | 2 | * | * | * | * | * | * | * | * | * |
|  | Georgia | 0 | 1 | * | * | * | * | * | * | * | * | * |
|  | Germany | 0 | 20 | * | * | * | * | * | * | * | * | * |
|  | Greece | 0 | 9 | * | * | * | * | * | * | * | * | * |
|  | India | 0 | 4 | * | * | * | * | * | * | * | * | * |
|  | Italy | 0 | 7 | * | * | * | * | * | * | * | * | * |
|  | Japan | 0 | 1 | * | * | * | * | * | * | * | * | * |
|  | Madeira | 0 | 1 | * | * | * | * | * | * | * | * | * |
|  | Maledives | 0 | 1 | * | * | * | * | * | * | * | * | * |
|  | Mongolia | 0 | 1 | * | * | * | * | * | * | * | * | * |
|  | Morocco | 0 | 1 | * | * | * | * | * | * | * | * | * |
|  | Niue | 0 | 2 | * | * | * | * | * | * | * | * | * |
|  | NZ | 91 | 154 | 37.8 | 67.1 | 178 | 3.98 | 9.52 | 15.8 | 43.3 | 537 | 533 |
|  | Poland | 1 | 4 | 14.8 | * | * | 14.8 | * | 14.8 | * | 14.8 | * |
|  | Portugal | 0 | 1 | * | * | * | * | * | * | * | * | * |
|  | Russia | 0 | 4 | * | * | * | * | * | * | * | * | * |
|  | S. America | 0 | 1 | * | * | * | * | * | * | * | * | * |
|  | South Africa | 0 | 1 | * | * | * | * | * | * | * | * | * |
|  | Spain | 0 | 3 | * | * | * | * | * | * | * | * | * |
|  | Turkey | 0 | 2 | * | * | * | * | * | * | * | * | * |
|  | UK | 0 | 5 | * | * | * | * | * | * | * | * | * |
|  | USA | 0 | 2 | * | * | * | * | * | * | * | * | * |
|  | Uzbekistan | 0 | 1 | * | * | * | * | * | * | * | * | * |
|  | Vietnam | 0 | 1 | * | * | * | * | * | * | * | * | * |
| **Pb** | Argentina | 0 | 1 | * | * | * | * | * | * | * | * | * |
|  | Australia | 1 | 2 | 582 | * | * | 582 | * | 582 | * | 582 | * |
|  | Austria | 1 | 1 | 43.1 | * | * | 43.1 | * | 43.1 | * | 43.1 | * |
|  | Belgium | 0 | 1 | * | * | * | * | * | * | * | * | * |
|  | Belize | 2 | 0 | 11.4 | 2.03 | 17.8 | 9.98 | * | 11.4 | * | 12.9 | 2.86 |
|  | Chile | 0 | 3 | * | * | * | * | * | * | * | * | * |
|  | China | 0 | 2 | * | * | * | * | * | * | * | * | * |
|  | Croatia | 0 | 1 | * | * | * | * | * | * | * | * | * |
|  | Denmark | 5 | 10 | 16.4 | 8.08 | 49.3 | 9.89 | 10.1 | 15.6 | 23.1 | 29.9 | 20 |
|  | Finland | 0 | 1 | * | * | * | * | * | * | * | * | * |
|  | France | 0 | 2 | * | * | * | * | * | * | * | * | * |
|  | Georgia | 0 | 1 | * | * | * | * | * | * | * | * | * |
|  | Germany | 4 | 16 | 11.8 | 1.81 | 15.4 | 9.49 | 9.92 | 12.2 | 13.2 | 13.2 | 3.75 |
|  | Greece | 3 | 6 | 15.5 | 3.39 | 21.9 | 13.1 | 13.1 | 14 | 19.4 | 19.4 | 6.29 |
|  | India | 2 | 2 | 18.8 | 4.14 | 22 | 15.9 | * | 18.8 | * | 21.8 | 5.86 |
|  | Italy | 3 | 4 | 17.9 | 13.6 | 76.2 | 9.68 | 9.68 | 10.3 | 33.6 | 33.6 | 23.9 |
|  | Japan | 0 | 1 | * | * | * | * | * | * | * | * | * |
|  | Madeira | 0 | 1 | * | * | * | * | * | * | * | * | * |
|  | Maledives | 1 | 0 | 17.4 | * | * | 17.4 | * | 17.4 | * | 17.4 | * |
|  | Mongolia | 1 | 0 | 10.6 | * | * | 10.6 | * | 10.6 | * | 10.6 | * |
|  | Morocco | 0 | 1 | * | * | * | * | * | * | * | * | * |
|  | Niue | 2 | 0 | 83.1 | 80.7 | 97.2 | 26 | * | 83.1 | * | 140 | 114 |
|  | NZ | 76 | 169 | 16.7 | 19.9 | 119 | 2.39 | 8.96 | 11.9 | 18.2 | 150 | 148 |
|  | Poland | 3 | 2 | 35.6 | 3.26 | 9.16 | 32.1 | 32.1 | 36.1 | 38.5 | 38.5 | 6.46 |
|  | Portugal | 1 | 0 | 11.4 | * | * | 11.4 | * | 11.4 | * | 11.4 | * |
|  | Russia | 1 | 3 | 10.5 | * | * | 10.5 | * | 10.5 | * | 10.5 | * |
|  | S. America | 1 | 0 | 13.6 | * | * | 13.6 | * | 13.6 | * | 13.6 | * |
|  | South Africa | 0 | 1 | * | * | * | * | * | * | * | * | * |
|  | Spain | 0 | 3 | * | * | * | * | * | * | * | * | * |
|  | Turkey | 0 | 2 | * | * | * | * | * | * | * | * | * |
|  | UK | 1 | 4 | 10.1 | * | * | 10.1 | * | 10.1 | * | 10.1 | * |
|  | USA | 1 | 1 | 10.1 | * | * | 10.1 | * | 10.1 | * | 10.1 | * |
|  | Uzbekistan | 0 | 1 | * | * | * | * | * | * | * | * | * |
|  | Vietnam | 1 | 0 | 18.2 | * | * | 18.2 | * | 18.2 | * | 18.2 | * |
