# Supplementary material for: Graphical Discrimination of New Zealand Honey from International Honey Using Elemental Analysis
Source: Biol Trace Elem Res. 2023 Apr 29;202(2):754–64. doi: 10.1007/s12011-023-03680-6 (PMC10764415; doi:10.1007/s12011-023-03680-6)
Supplement: Supplementary file 4 — Supplementary file4 (DOCX 390 KB) [file 12011_2023_3680_MOESM4_ESM.docx]

**Graphical discrimination of New Zealand honey from international honey using elemental analysis**

Megan N. C. Grainger^a*^, Hannah Klaus^a^ Nyssa Hewitt^a^, Han Gan^b^, Amanda D. French^a^,

*^a^ School of Science, University of Waikato, Private Bag 3105, Hamilton 3240, New Zealand*

*^b^ Department of Mathematics, University of Waikato, Private Bag 3105, Hamilton 3240, New Zealand*

*Corresponding author: [megan.grainger@waikato.ac.nz](about:blank); +64 7 837 9621


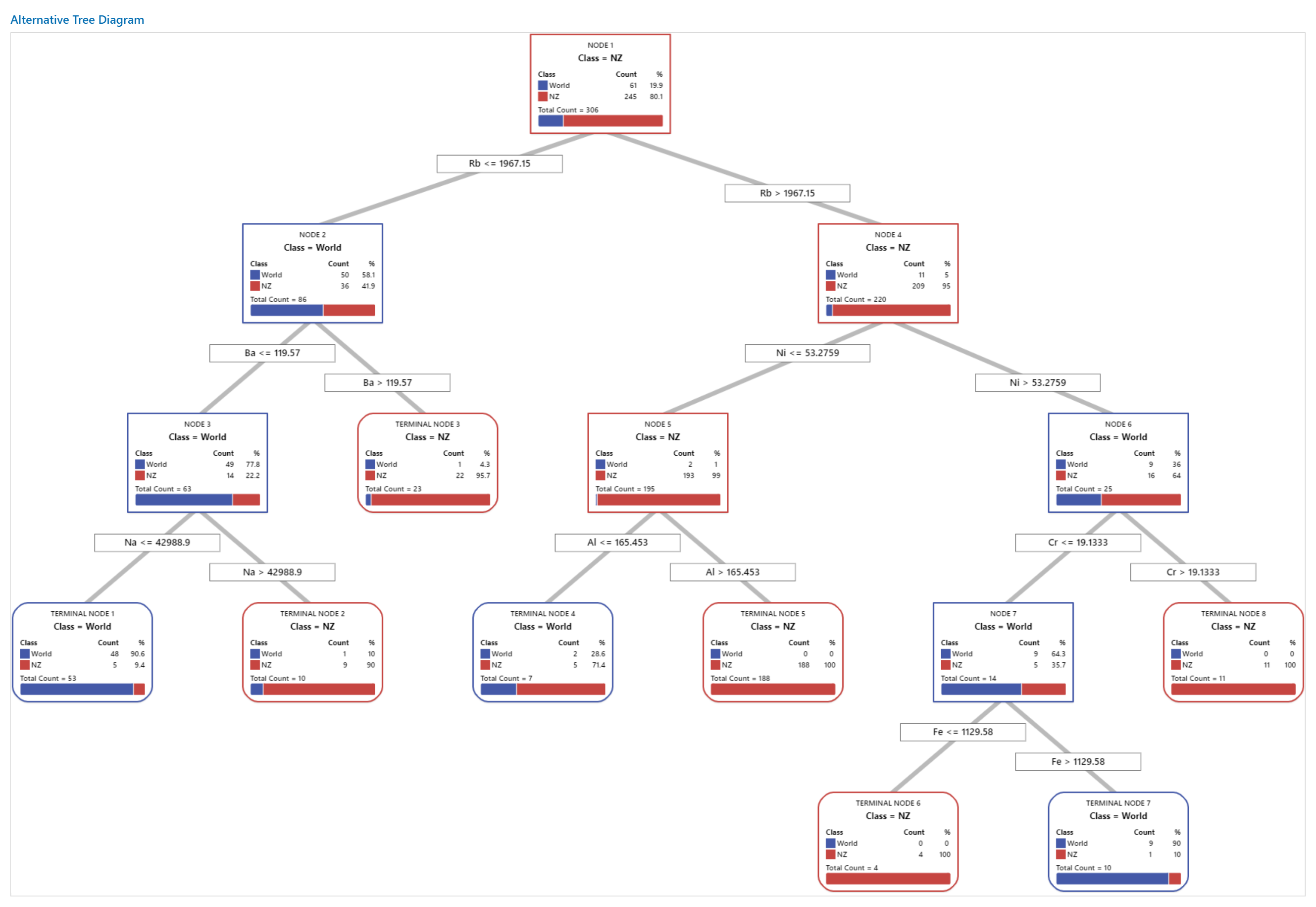


**Supplementary Information S4** Decision tree with 10 terminal nodes for New Zealand and Europe (*n* = 306) to produce the lowest misclassification cost (0.1880).
